# Large observational bias on discharge in the Indus River since 1970s

**DOI:** 10.1038/s41598-018-35600-3

**Published:** 2018-11-23

**Authors:** Jingshi Liu, Shichang Kang, Kenneth Hewitt, Linjin Hu, Li Xianyu

**Affiliations:** 1 Key Laboratory of Tibetan Environment Changes and Land Surface Processes, Institute of Tibetan Plateau Research, Chinese Academy of Sciences, Beijing, China; 2 State key laboratory of cryospheric sciences, Northwest Institute of Eco-environment and Resources, Chinese Academy of Sciences(CAS), Lanzhou, China; 3 CAS Center for Excellence in Tibetan Plateau Earth Sciences, Beijing, China; 4 Cold region research center, Wilfird Laurier University, Waterloo, Ontario N2L 3C5, Canada; 5 Hydrology and water resources bureau of Xinjiang, Urumqi, Xinjiang China; 6 Hydrology and water resources bureau of the Yangtze River, Wuhan, Hubei, China

## Abstract

The discharge of one of the world’s largest river - Indus River was reported to be increasing that was not supported by the Karakoram (KK) glacier expansion. A major hydrometric bias was ignored, which seemed similar to the montage that the Himalayan glaciers would disappear. This study proposed a framework for quantifying the bias resulting from inaccurate data affecting hydrologic studies on the Indus. We constructed a statistical model by converting the rating curves of rivers into air temperature (T) – discharge (Q) curves from an adjacent catchment in China where flow measurement was carried out using a standard method. We found that most flow data for the Indus were much greater than the error limits of T-Q curves estimated by daily data, a greater bias occurred in recent decades when discharge increased, the higher the flow was, the larger the bias was. The estimated mean annual and maximum monthly bias was 22.5% and 210%, respectively. These biases indicated that discharge increase in the Indus probably resulted from the large errors of hydrometrics without a scientific basis. We suggested a montage bias was needed in the hydrologic science of KK’s rivers that may strongly affect water resource management.

## Introduction

The high-altitude terrains of the Himalaya (HL), Karakoram (KK), Hindu Kush and the adjoining Tibetan Plateau (HKT) contain some of the largest glaciers and most extensive snow-covered areas in the world outside the polar regions^1–7^. Several of the world’s largest and most heavily populated river basins, such as the Indus, Brahmaputra and Ganga, originate in these regions. Snow and glaciers provide significant amounts of water for multipurpose usage, including agricultural irrigation, water supply, and hydropower generation, to a vast population of South Asia. Any substantial hydrologic response to climate change, such as reduced glacier areas or increases in river flow, can have profound impacts on water resources management^6^. The retreat of the Himalayan glaciers has already become an urgent concern for scientific inquiries^8–18^. The Indus River, the 12^th^ largest water resources in the world, is a critical factor in the lives and economies of more than 260 million people in Pakistan, India and Afghanistan. It supports one of the largest irrigation systems in the world and is also the source of extreme flood disasters^7,19^. The upper Indus River especially has a history of the major earthquake and landslide disasters^6^.

**Figure 1:**
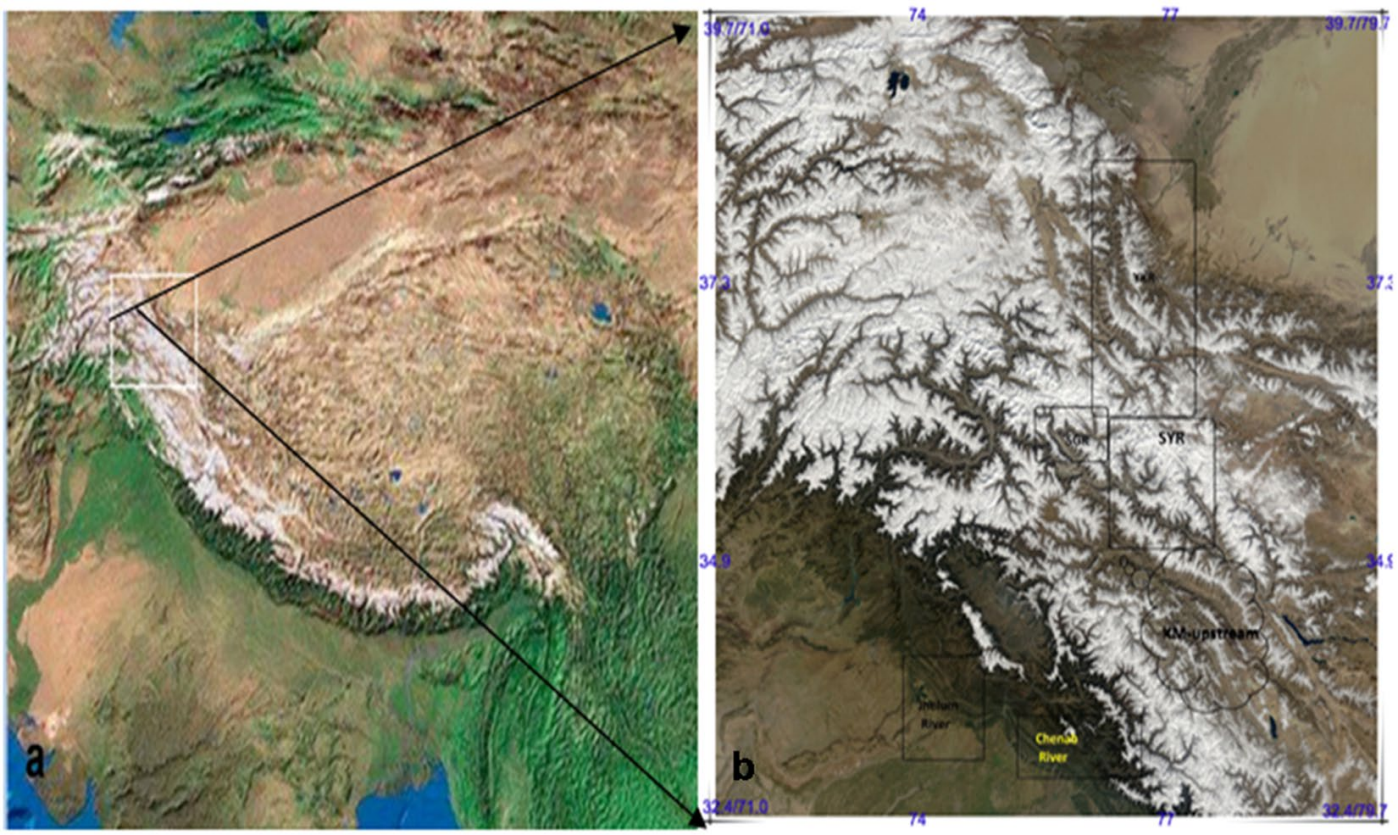
Location map of studied area and basins. Geospatial data of the base map for the image using a free encyclopedia at https://en.wikipedia.org/wiki/high-mountain_Aisa is covered by a CC-BY SA license at https://creativecommons.org/licenses/by-sa/4.0/deed.en, and the b image coded with Tajik- istan_A2004296 _ 0605_1 km at http://www.image.nasa.gov. credits to Jacques Descloitres, MODIS Rapid Response Team, NASA/GSFC. Both graphs were created by Liu using free XiuXiu_ Preview v5.1.0. (http://xiuxiu.meitu.com). The block with black letters represents the basin profile and the river name. (In the text, YKR- the Yarkand River; SGR-Shigar River; SYR-Shyok River; KM- the Indus above Kharmong station; KC- the Indus above Kachura station near the SGR).

The Upper Indus River (UIR) originates in two great mountain ranges, the KK and Northwest HL, while its Kabul branch drains the Hindu Kush Mts. (Fig. 1a). According to the Glacier Inventory of Pakistan, there are 11,413 glaciers covering an area of 15,062 km^2^. It is also the site of some of the largest glaciers in High Asia, such as Siachin (Area 1400 km^2^), the fourteen largest comprise about 75% of the entire ice cover. The UIR cryosphere also includes a somewhat larger region of mountain permafrost, several thousand rock glaciers, and even larger areas affected by periglacial processes. Future climate change is expected to affect all these components of the hydrologic cycle^20–27^, and the cold and arid environment of north Pakistan and northwest India^28–31^.

The glaciers act like water towers transferring a large amount of meltwater into the Indus (Fig. 1b), with an annual runoff of 2339 × 10^8^ m^3^ at the Kachura (KC) station contributing to the large dam at Tarbela. Considering the high altitude and river dynamics of the Indus, particularly upstream, the reliability of hydrometry has received international attention^7,32^. Various studies have reported increased discharge for the UIR from 1985 to 2010^24–31^. It is considered to be associated mainly with the ablation season of the glaciers and has the potential to create a singular threat for Pakistan. Some even blamed the great floods of 2010 on glacier melting – although that was a mistake^19^, the main trigger was heavy rainfall across the northern part of the basin. In contrast, however, the water variability in the glacier-fed Yarkand River (YKR) in the Tarim Basin of China is in a stable condition. This river also drains from part of the KK range, immediately north of the UIR. It is puzzling why this should be so, and there is an urgent need for reliable quantitative estimates of the relative contributions of ice, snow and rainfall to these rivers. If these are available, the hydrologic effects of two main concerns of climate change, global warming and changes in annual precipitation patterns, can be ascertained with greater certainty. This is the main purpose of this paper.

To date, the increase of the Indus flow has been accepted without a critical statistical analysis of potential flow errors. Its flow has been predicted to increase under global warming, whereas observed changes in glaciers since the 1990s, some even say 1970s, involve thickening and advance of glaciers in the highest parts of the Karakoram Range, and little or no change in total area and mass, according to both glaciological observations and satellite images^12,29^. Some investigations suggest a slight mass gain in recent decades^33^. However, these assessments also do not take into account uncertainties and differences in water balance and relative contributions of rainfall, snow and ice of rivers in the region. These can be explored firstly by comparing the UIR and YKR. Two basic considerations are the glacier coverage, which is large in both cases and certain similarities in climate, both flow through the strongly glaciated region in the world with high elevation, deep valleys and river sediments. These rivers are known in the scientific community for their unique hydrogeological impacts as well as their hydrometrics and value as model systems^32–38^.

The Indus and the YKR show considerable similarity in their responses to the Indian monsoon in summer and the westerly winds in winter and spring^7,34^. Factors that strongly affect the hydrodynamics of the meltwater rivers are shown in Fig. 1b. Recent studies by Biswajit *et al*.^2,3,28,29^, for example, found an unbalanced river flow at the KC, the main stem of the UIR that receives flow from the Shigar (SGR) and Shyok (SYR) Rivers, upstream of Khamong (KM) and in a small unguage (2.1%) between the KC and KM, in which 10–20% of the unbalanced flow in winter and spring was ambiguous because the sources were unknown (see S1). The related studies clearly show that the adjacent SGR and SYR have anomalous hydrologic characteristics even though they have high glacial coverage, particularly in the SYR (Table 1). However, the annual runoff of the SYR is only one-third (325 to 920 mm) of the SGR. Moreover, the meltwater flow of the SYR in July and August is only twice as large as that of the SGR. Nevertheless, the glacial and drainage areas of the SYR are 3.5 and 4.5 times larger than those of the SGR, respectively. In addition, the average annual and monthly flows at the KC receive unbalanced inflows from the SGR, SYR, KM and the non-gauge for the same records for 12 years (1985–98, except in 1993 and 1995), particularly in Jan., May and Oct. The extra flow is approximately 40% larger than the monthly average at the KC (see S1). However, the authors did not explain these biases. The measurement error of a river flow is proportional to the river scale; the greater the flow, the higher the error. Internationally, the accepted hydrometric error of a large river is <15% in Europe^35–37^ and China^38^. We challenge the previous authors’ conclusions that the recent increase of river flow in the UIR matches the pattern observed in previous studies. We also challenge the various scenarios of extreme floods and the water resources projected by the previous authors’ hydrological models, which were based on inaccurate data of daily and monthly discharge.

**Table 1:** Hydro-glaciological parameters.

River Name	Station Name	W-station (m, asl)	Area (km^2^)	G-area (km^2^)	G-terminus (m, asl)	Runoff (mm)
Shigar	Shigar	Skardu/2220	7040	2121	2720	923
Shyok	Yogu	Skardu/2220	33154	7696	2800	325
KM-stream	Khamong	Leh/3540	70400	2523	3580	217
DK-stream	Dumkar	Leh/3540	61470	2032	2850	177
Kachura	Kachura	Skardu/2220	114079	11692	2720	273
Jhelum	Mangla	Mangla/384	33870	148/snow	3410	856
Chenab	Marala	Marala/650	26035	2350/snow	3130	1155
Yarkand	Langan	LG/2000	32880	4896	3800	168

Station is name of th gauge; W-station means weather station and its altitude;

Area means drainage area of the basin; G-area means glacier area in the basin;

G-terminus means the lowest altitude of glaciers in the basin; Runoff means the average runoff.

## Results

**Figure 2:**
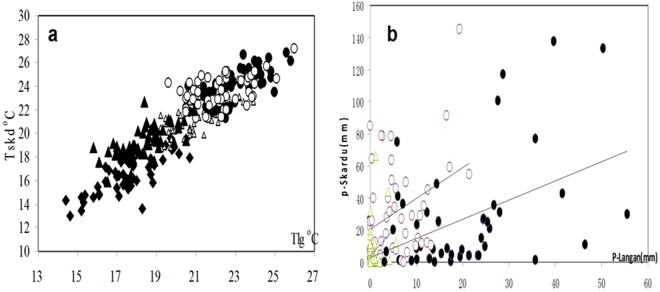
(**a**) Teleconnections of monthly air temperature T (May-Sep., ◆, ○, ●, ▲, △) and precipitation R in melting period between SKD and LG during 1961–2011 (p < 0.001). Teleconnections among monthly air temperature T (Tmay (◆), R² = 0.65; Tjun. (○), R² = 0.52; Tjul (●), R² = 0.53; Taug.(▲), R² = 0.25; Tsep. (△), R² = 0.49) (p < 0.001) and (**b**) Teleconnections among monthly precipitation P in spring and autumn (Rapr., R² = 0.13; Rmay(●), R² = 0.20; Rnov., R² = 0.09) (p < 0.01) between SKD and LG during1961–2011.

According to the same pattern of both regional climates in the KK rivers, we explored the teleconnections of monthly air temperature and precipitation in the summer melt period (May.-Sep.) between the LG station in YKR and the Skardu station in the Indus. For 50 years, there is a strong correlation or similarity of the temperature and a weak correlation in precipitation (0.16–0.52, p < 0.05) between the two areas (0.24–0.7, p < 0.01) which are shown in Fig. 2 and the unreliable correlations between their discharge appear in YKR and the Indus (see the SI2).

**Figure 3:**
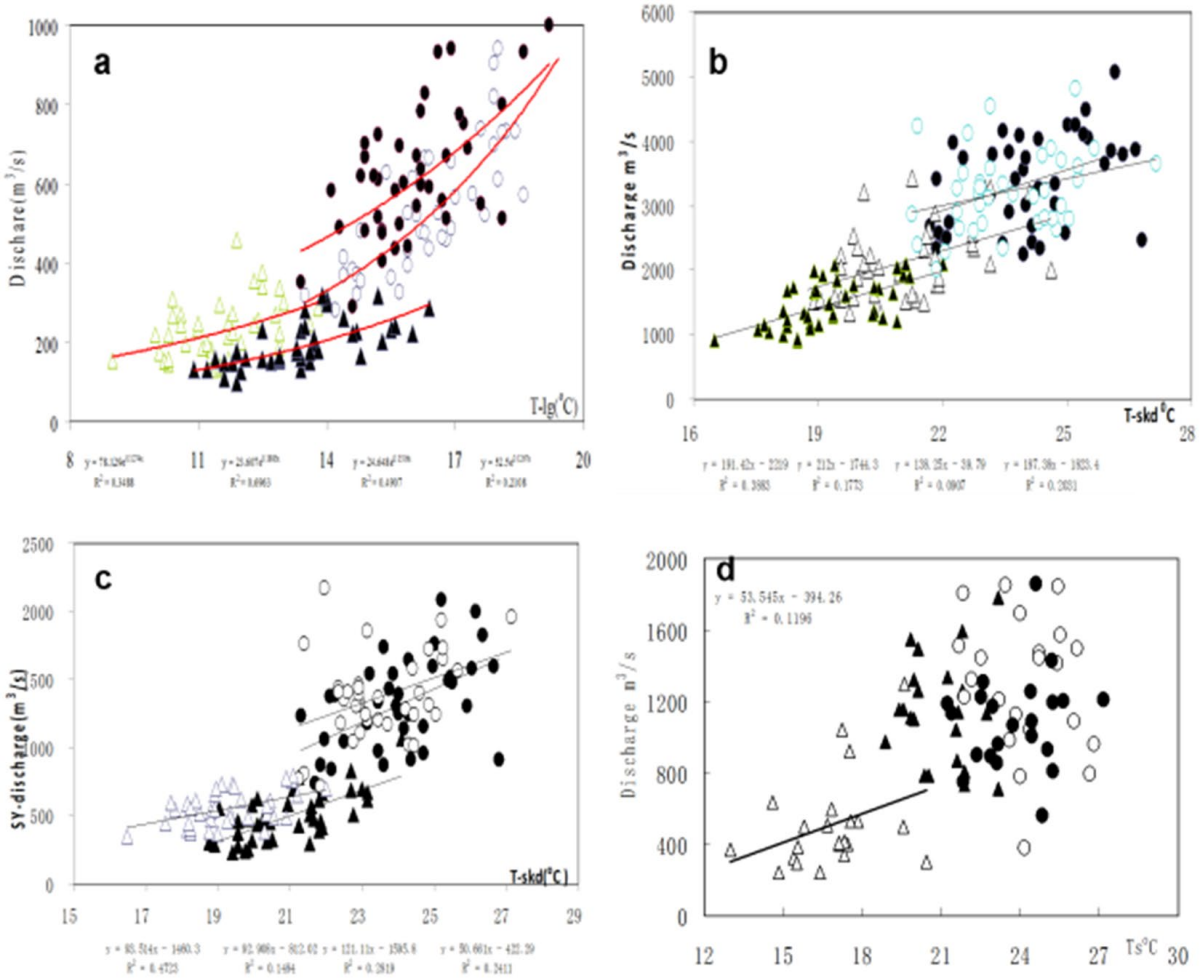
Monthly correlation between the T and Q during Jun. to Sep. in YKR (**a**) (40 yr., R = 0.38, P < 0.01), KC (**b**) (38 yr, R = 0.38, P < 0.1), SYR (**c**) (38 yr, R = 0.47, P < 0.05), ▲-Jun, ●-Jul, ○-Aug, △-Sep. Graphs for the KM (**d**), (22 yr, R = 0.33, P < 0.1). The graph for SGR and DK see SI5.

As previously documented by snow-ice hydrology, a basic model is represented by a positive correlation between melt discharge (Q) and air temperature (T)^8,9^. Figure 3a illustrates the close correlation between monthly T and Q in summer in the YKR, where R^2^ is >0.32 from Jun. to Aug. With the same model, we expect that there should be a closer correlation in the adjacent sub-basins of the Indus because they have higher snow and ice coverage and meltwater in the warm (hot- dry) seasons. Thus, we calculated the correlation between monthly T and Q, and they were positively correlated for the summer data at a significance level of 0.05 or better. Most points of the T-Q scattered in a tail or two limbs, resulting in no correlation, which implies that there are more than 2 flows at the same T, as shown in Fig. 3b–d. There was a strong correlation in Jun. and a weak one in Jul. at both the SGR and KC, but none in Aug. (3b, c), when the Q values were too large to relate to the T. The Q was strongly related to the T in Jun. and Sep. and weakly in Jul. for the KC, but not in Aug. (3b), when the Q was too chaotic to be related to the T. The Q was weakly related to the T in May for the KM, but no correlation in Jun.-Aug. (3d). A weak correlation was found in Jun. and Aug., but none in Jul. in the DK (SI2), when the Q was too scattered to be related to the T. Consequently, the R^2^ was in decline from May to Aug. in the KC and SYR, where Q was not positively correlated with the T for snow-ice hydrology. This finding implies that there is a great uncertainty in the hydrometrics of the UIR flow (see SI2 for monthly correlation). We conclude that the poor and lacking correlations are the results of poor measurements of daily Q.

To examine the quality of the Q data in the UIR using the methods (1–4) proposed in this study, we cross-tested the magnitudes of both daily and monthly Q (m^3^/s) for 12 years between the large SYR and the small SGR, the small SYR and the large KM or KC, and the large KM and the small DK because they are all closely adjacent, as shown in Fig. 1b. The Q of the SGR should always be smaller than that of the SYR; otherwise, the data can be considered relatively unreliable. Unfortunately, in 12 years, we found that there were a great number of irrational monthly Q-values in the SGR that were greater than those of the SYR. Among them, there were 7 in May and Jun., 1 in Jul. and 2 in Sep., respectively. The worst case was that the first Q of the SYR on 1^st^ Jan. 1981 abruptly jumped to 177 from the base flow of 47 at the end of Dec. 1980, then increased by 277%, which is impossible in the coldest Jan., at that time, T was far below 0 °C. The mistake not only caused the mean Q of the winter and spring of 1981 to be 200% greater than the annual mean value but also resulted in the erratic data of the following day, month and year, such as annual mean increased for 38 years. For detailed results, see SI3. The abnormal jump and drop of the Q also frequently occurred in the SYR and the KC (details in see SI3). For the anomaly in the DK of India, monthly Q was 52.5 in Mar.-Apr. 1995 and 47.7 in winter-spring 2001. Detailed results in 1987-88, 1990 and 1992 can be found in SI3. The worst anomalies imply that the DK station in India is the most poorly operated national hydrometric station.

To explore the monthly T-Q model, we found that there was not only no correlation between the T and Q (m^3^/s) in summer in the KM and DK but the mean Q of Jun. was also greater than that of Aug. and Sep. in the 22-year data (see SI4). Particularly in 1990, the mean Q declined from 1783 in Jun. to 1197 in Aug. in the KM and from 3180 in Jun. to 897 in Sep. in the KC in 2008. The Q in Jun. must be smaller than that in Jul. and Aug. in these meltwater -fed basins because the T in Jul.-Aug. is always higher than in Jun. This inversion never occurred in the other 3 stations, especially in the YKR. This pattern implies that Q in Jun. and the erratic estimate in Jul. and Aug in the KM are large in error. In addition, the mean Q of Jun. is also much greater than that of Sep. in the KM and the KC, and these regimes can be seen in the SGR, SYR and YKR as well as in Fig. 2 (also in SI4 -Fig. 3).

**Figure 4:**
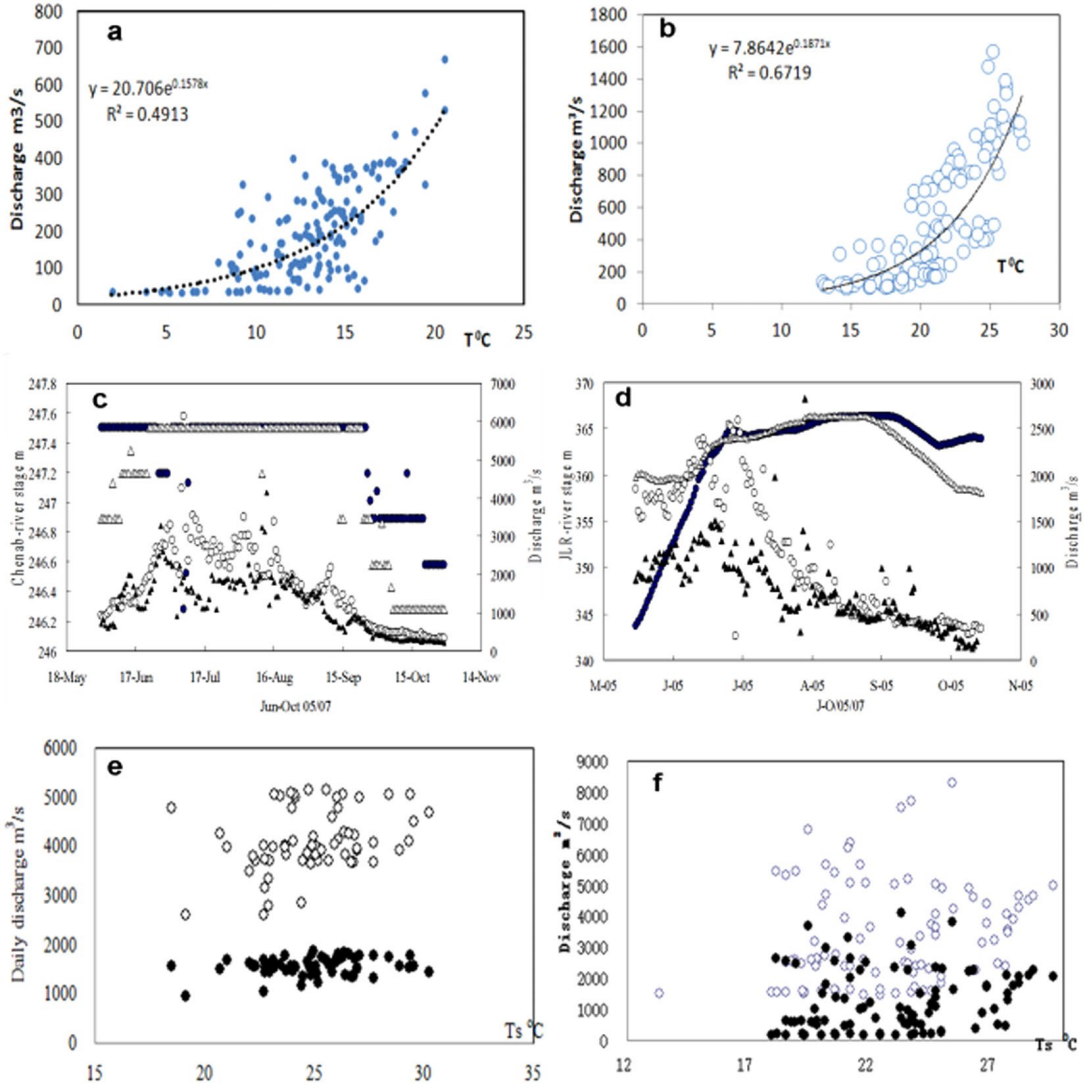
Hydrograph of the summer daily discharge and air temperature (●/○) for the YKR (**a**,**b**), for the KC (○ in **e**,**f**) and SYR (● in **e**,**f**), daily river stage (△/▲) and discharge (●/○) for the Chenab (**c**) and Jhelum (**d**) during May to Oct. in 2005/2007, many unchanging stages in the two basins in the high flow periods imply not only without continuous observation of the stage for long time, but also the highly changing discharges were not estimated from the real stages. The high flow for the KC (○) and SYR (●) is in 2006 and 2010, the correlation with a lag of 2d. Similar to 3a, the plots in either random or a two-limbs or displaying without any a correlation in high water all are beyond big bias of previous flow process, the larger a flood, the larger the points irregularly distribute. The largest flood in 2010 for example, the largest Q was not attributed to the highest T in Jul. and Sep. (the graph for SGR see SI4-Fig. 2).

Why did the poor monthly T-Q curve occur in the Indus? In fact, the daily Q should be related to the daily T in the form of a T-Q curve if Q is accurate, especially with increasing T. Thus, we examined the daily correlation between the T, regarded as the river stages were partly available in the JLR and CNR only, and the Q for the data periods in the 4 stations of the YKR, SGR, SYR and KC at the significance level of p < 0.05 (30/31d). It is clear that the stable and close correlations are found in the YKR in both high and low flow years (Fig. 4a,b). However, we found many erratic Q data when most points of the T-Q were in a tail or two and a few limbs (in groups) in the SYR and KC of the UIR as shown in the Fig. 4e,f, the worst was that there were no rating curve in form of daily river stage (meter) to the Q for the JLR and CNR because many unchanging stages in the high flow periods imply not only without continuous observation of the stage for a long time but also the highly changing Q were not estimated from the real stages in Fig. 4c,d, which implies that there are at least more than 2 Q at the same T or the stage, as shown in Fig. 4c, even without any T-Q curve. For the SI4-Fig. 2, there were clear erratic points between the daily stages and the Q when the stages were increasing while the Q declined, all extreme (the highest and lowest) stages did not respond to the extreme (maximum/minimum) Q. The daily stage drop to the lowest 317 m from 343 m in the JLR, as the same, the stage jumped to the highest 365 m from 247 m in the CNR. The daily Q jumped to the maximum 2974 m^3^/s from 340 m^3^/s in Dec. 2006 in the JLR, the Q jumped to the maximum 12288 m^3^/s from 246 m^3^/s in Jan. 2008 in the CNR.

The best monthly correlation between the Q and the T at the SGR during May to Sep. is a polynomial function, as shown in Fig. 3, and then the Q in Jul. and Aug. can be interpolated and extrapolated using the T-Q correlation inputted by the T in m^3^/s. The interpolated results implied that the average bias of monthly Q was −14.9% in May, no bias in Jun., and it was 5.7% and 16.5% in Jul. and Aug., respectively. The maximum bias was −280%, which occurred in May 1990. Similarly, the bias of monthly Q of the SYR was −54.9% in May but was 9.2%, 10.6% and 14.7% during Jun. to Aug., respectively. The maximum bias of 164% and 100% occurred in Jun. 2010 and Aug. 1990, respectively. The rough estimates suggested that the Q was biased during Jun. to Aug. by an average of 55.6%, 42% and 22% in the KM (SI6-table), respectively. However, in the DK, the values were −12% and −9% in Jun. and Jul., and 8% and 24% in Aug. and Sep., respectively. Similarly, the results for the KC (Fig. 4e) suggested that the Q from May to Aug. were biased by an average of 19.6%, 13.1%, 6.3% and 7.2%, respectively, and the maximum bias was 39.6% in Jun. 1996 and 33.4% in Jul. 1989, respectively. Finally, the average in the summer in the KC was biased by 11.6%.

Due to very dangerous and difficult conditions, it is nearly impossible to directly measure the highest annual flood flows in the UIR. The extremely fast flow peaks at night are easily miscalculated. In any case, these are based only on river stage in certain rare instances, and the higher the flows the less reliable is stage-to-discharge rating. The meltwater flood is usually attributed either to the early warmest weather in Jul. or Aug. or to uncommon storm rain. There should be consistency in the date of annual flood occurrence of the river network. With the data of annual daily flood (m^3^/s) for 12 years at the SGR, SYR and KC (see SI5), the 1986 floods in both the SGR and KC occurred on Aug. 3^rd^ and 5^th^. However, it occurred on Aug. 20^th^ in the SYR, whose discharge suddenly jumped to 2010 from 1210 with a lower temperature than that in early Aug. Similarly, the 1987 floods in both the SYR and KC appeared on Jul. 25^th^, but occurred on Aug. 24^th^ in the SGR, which was almost 1 month earlier or later than the actual date. The same errors occurred in 5 out of 12 years with detailed results in 1999, 2006 and 2007 (see SI3 and SI5). In summary, the increasing flood volume of the UIR, due to lack of data on the date and extreme weather, is particularly susceptible to higher bias than that of the early melt flood.

The Indus is a critical source of water, and most of the flow is derived from melting snow and ice. Hence, the summer Q is strongly correlated with winter-spring snowfall and summer T, although the estimated Q links may operate in the opposing directions in glacier-fed and snow-fed hydrologic regimes. From 1961 to 1999, there were significant increases in winter, summer and annual precipitation, and warming occurred in winter, whereas summer exhibited a cooling trend^9^. These trends will impact water resource availability. However, comparing the monthly T and Q rating curves between the YKR and the SYR reveals that they are north-south neighbors in the K2 with almost the same drainage area, glacial area, and elevation. There are stable curves and higher accuracy of Q from the YKR because Chinese hydrologic engineers routinely calculate the manually corrected automatic river stage for 24 hours x 365 days, and then jointly calculate the multi-point velocity of 36–40 times per year to estimate daily discharge in summer (May-Sep.). In addition, in China, at least 3 observers are required to determine and correct the rating curves, and we seldom replace observers because a long-term professional experience is very important for managing such large and dangerous rivers with frequent outburst floods. Thus, the correlation of the T-Q in the YKR is much better than that of the UIR (Fig. 3). In the UIR, the correlation becomes weaker and weaker with increasing ablation from May to Aug., even without replacing the rating curves in the high- energy with T and the meltwater to generate higher flow in the warmest and driest Aug. It can be concluded that great bias comes from incorporating abundant erroneous Q values into the T-Q curve, corrupting the correlation between the T and Q in the UIR.

With the great number of errors suggested as above, the errors have propagated and amplified the subsequent discharge and annual flood estimations based on the theory of geometric error propagation (see SI6). The extreme anomalies, e.g., from winter to early summer in 1981, gradually elevated the annual mean monthly Q by at least 5.4%, 5.5%, 6.3% 11.1%, 11.9% and 9.5%, respectively, which is the predominant factor contributing to the so-called increased water of the SYR. Similarly, the monthly Q caused by starting with a large error in May 1996, was elevated by 39.4%, 10.5%, 8.7% 7.9% and 19.4%, respectively, from May to Sep. Then, the total increase in annual mean was an extra 7.3%, which is the dominant contribution to the so-called meltwater increase of the KC. Again, the anomaly with the smallest Q in Jul. 1984 in the KM reduced the mean value of Jul. by 3.4%, whereas the largest Q in May and Jul. in the DK elevated the mean value by 23.3% and 7.8%, respectively.

With monthly data from the same period in the melt season (Apr.-Sep.) for the SGR, SYR, KM and KC, the ungauged flow can be approximately estimated using the area ratio of 2.1% between the sub-basin and the KC (see SI7). Then, we can simply balance the water budget of the UIR using Equation 1. The results showed a net positive budget in every year (see SI7). Mean monthly budget was 10.2%, 19.6%, 13.1%, 6.3%, 7.2%, 11.3% and 11.5% from Apr. to Sep., respectively, generating a positive 11.5% in annual melt flow. The greatest monthly budgets were 33.9% (1988), 39.4% (1989), 33.4% (1989), 21.7% (1996), 17.6% (1989) and 31.6% (1990) from Apr. to Sep. The greatest annual imbalance was 22.1% in 1989. Another extreme imbalance was between the SYR and KC in 1981. The SYR’s monthly Q (m^3^/s) during Jan. to Jun. was 166/141, 158/121, 151/140, 243/203, 663/548 and 2350/1532, respectively, which was even greater than the KC excluding inflows from the SGR, the KM, and the ungauged areas. These unrealistic data led to an uncommon and huge negative balance of −53.4% in Jun. at the KC. It is clear that the data in the SYR have been exaggerated. Therefore, the greater bias is expected to support the irrational hypothesis of the so-called warming and greater melting in the UIR.

**Figure 5:**
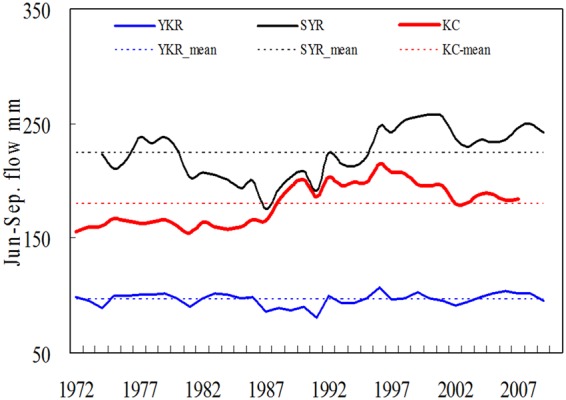
Long term variation of the summer (Jun. to Sep.) flow with a 5 years moving mean in the YKR, SYR and KC stream. A significant upward trend is in the UIR flow, but insignificance is in the YKR. The KC flow shows a decline then a rise before and after 1986, but the SYR shows a stable before 1986 then a rise and slight decline despite the SYR is the greatest contributor to KC.

The data suggested that meltwater significantly increased in the UIR from the end of 1980, as shown in Fig. 5. Even early in 1987, it increased by an average of 19% in the KC (19.2%/15.8%/22.8%, Jun/Jul/Aug.) and increased by 15.5% in the SYR (10.4%/12.4%/18.5%, Jun-Aug.), but insignificantly rose by 2.1% in the YKR. However, the T at Skardu or Leh did not rise in Jul. and even slightly cooled in Aug. and Sep. Although the KC’s flow remarkably increased, it did not increase in the YKR, particularly in Jul. These data provide strong evidence that the increase cannot be attributed to the so-called warming. It is not by chance that the greatest bias in the Q has occurred since 1990. The greatest bias, for example, occurred in 1994 in the KC and the SYR and in 1990 in the KM and the DK. The biased water data are responsible for the increased water resources in the UIR.

## Discussion

As is well accounted for in the national standards of hydrometrics, it is critical to obtain accurate data on river stage, riverbed and velocity profile. The greater the variability in river stage, the more frequent measurements are needed. In addition, accurate velocity relies on riverbed depth, so that the deeper the river flow, the more velocity measurements are needed to obtain the average velocity. Collecting these data is very dangerous and laborious intensive. Given that river Q is usually obtained according to the so-called rating curve, a number of different sources of error affect the derived observations. Detailed studies have been conducted on the Po River (Italy) and on the YKR according to the European^35–37^ and Chinese standard of hydrometrics^38^. The studied sources of error include errors from river stage and current velocity, discharge which is utilized to parameterize the rating curve, interpolating and extrapolating the error of a rating curve, the presence of unsteady flow and so on, particularly the absence of nighttime data. Variability in the dynamic response of river stage in the Indus also relates to the shape of riverbed and surface hypsometry. To find the error source, we investigated hydrometric sections and used instruments at the stations. The section widths were approximately 110 to 140 m at the KC, YG and SG and 70 to 80 m at the KM and DK. All river flow depths in high flows were greater than 10 m (see SI7 and 8). Both Pakistan-WAPDA and India-DHEP observed the river stage during the day (9 am to 5 pm), which can lead to an uncertainty of 66.7% on daily averaged river stage (16 h, 6 pm to 8 am). Furthermore, they observed the flow velocity only 12–15 times per year, not every month, especially at the highest river stage related to the annual floods, e.g., in 2010^19^.

**Figure 6:**
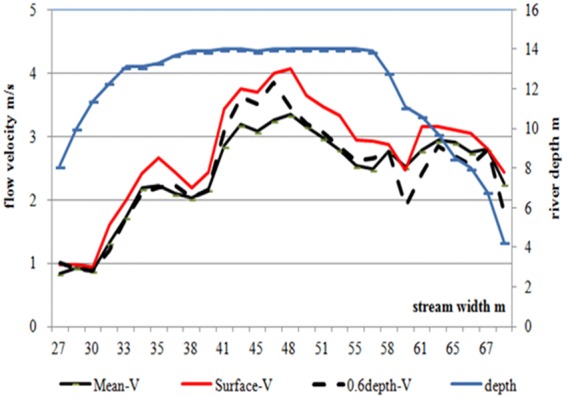
Velocity and depth profiles of the river flow at Jhelum River, Pakistan in Aug. 2014. The depth-velocity meter was Rio-G 600 KHz of the ADCP, the measurement was driven by a boat to transit the metric section of 52 m in width and 4 to 14.5 m in depth of metric profile. There were 168 data of the river depth and 140 data of the velocity using 6 point velocity (at 0.3 m, 0.2, 0.4, 0.6, 0.8 of the deepest depth at every section, respectively) in every 2 m along riverbed, It is clear that the metric section is in a shape of U with the deepest 14.3 m (blue line) at the current center, the highest and mean (red/black line) velocity is 4.10 m/s and 2.5 m/s at the surface and below the 0.6 depth of the flow, respectively. The estimated discharges would have +60% bias if using the surface velocity than that the mean. The ranging velocity between was 4.1 and 2.5 m/s indicating in extremely unsteady flow.

The greatest daily discharge that was extrapolated by few data on the velocity likely led to huge biases. Unfortunately, they used a floating or cable-sinker (SI7-Fig. 2) to measure both water depth and surface velocity on the flood days. Thus, large errors were also expected when estimating daily mean river stage and velocity from only the daytime data because two limbs are formed by the meltwater daily cycle (following T) of the stage with an increasing (erosion) and then a falling curve (deposits sediment) flow. However, the cable-sinker carrying the velocity meter must sit vertically on the riverbed; otherwise, flow depth and velocity will be over-measured. The cable velocity meter cannot be placed vertically on the riverbed under the frequent unsteady flow in the KK rivers. Thus, the meter is also placed at incorrect depths to generate the incorrect average velocity of the section because real and detailed information on hydrometrics is not available from the WAPDA. To find the error range of the discharge measurement in the Pakistani rivers, we observed a few floods with our ADCP metrics on the Jhelum River in Aug. 2014. We found that both flow depth and velocity of the river were 60% over-measured by the WAPDA using the fish-meter with one point velocity at the water surface (at 0.3 m depth) versus our multi-points as shown in Fig. 6. In addition, it is impossible to measure both the extreme depth and fast velocity of a high flow that is faster than 4.0 m/s due to the limitations of the manual current meters in the UIR.

The metric depths at metric sections of the KC, SYR (Yugo), KM, DK, Jhelum and Chenab usually ranged from 10 to 15 m in annual flood, we did not believe the huge shifts (>100 m) of the river stages of JLR and CNR in Fig. 3c,d. Therefore, one error might come from the mean velocity if based solely on the surface flow. The deeper a river flows, the more velocity measurements are needed to obtain an average velocity at the vertical depth. WAPDA measured the discharge with the float and cable-sinker meter at all stations. The velocity meter is very difficult to set at the accurate position along a depth profile because of the huge shock of a large flood. In addition, the KK riverbeds are characterized by a U shape with narrow and deep riverbed and many huge rocks that frequently deposit and flood the sediments between the high and low flow, altering the water depth.

Not only is the meltwater variability in the YKR greatly different from that of the UIR, but so are the western Hunza and southern Astore of Pakistan and the Sutlej, an Indus tributary. Archer *et al*. reported that there was an erratic relationship between both summer T and Q and rain in the UIB^10^. The Q increased, but the T declined^11,12^. With distinct hydrologic regimes in summer, the volume decreased by 20% and 57%, respectively, which was governed by the ablation of glacier, permanent snow and monsoon rainfall. There was slightly declining summer runoff in the Hunza^13,32^, Sutlej^15^ and the Astore^17^, which was estimated to have been resulted from the observed 1 °C fall in mean summer temperature since the 1980s to the mid-1990s with even greater reduction in summer. The observed downward trend in summer T and runoff was consistent with the observed thickening and expansion of the Karakoram glaciers, in contrast to widespread decay and retreat in the eastern Himalaya. Finally, the large numbers of surge-type glaciers, with sudden large advances and extended periods of retreat and stagnation, were not in phase with climate. In fact, most large glaciers have several surge-type tributaries that upset their mass balance relations and terminus fluctuations^14^. Glacial surges and related outburst floods have frequently occurred in the Karakoram^7,34^ since the mid-1990s despite rising temperature in the Karakoram.

We suggest that the apparent increase of the discharge from the UIR is an erroneous outcome of measurement, estimation or and modeling errors for meltwater and annual flood. We strongly suggest that the WAPDA of Pakistan and the India-NHEP improve their methods on hydrometrics as that as the guide of WMO^39^, particularly on river stage, velocity meter and river depth of the large rivers. In sum, the temporally linear, spatially homogeneous concepts of hydrology change and glacial characteristics have restricted applicability. In contrast, the real world of glaciers and their environment may be likened to a photographic montage in which each montage element itself is a complex and dynamic picture^16^. The same situation might exist in the Himalayan river, such as in Afghanistan, Nepal and Bhutan. This issue is a great challenge and cannot be ignored by water resources management in the future.

Our results seem to reject previous studies that computed and predicted the water resource variability in the UIR, without testing the reliability of discharge data. We propose that a great bias in estimating river flow, due to the strong unsteady high flows in the highly irregular valleys, aggravated by the alternation of steep gorge sections and broad basins with extensive intermontane sedimentation. This study also has implications for the glaciological, flood and sediment transport processes of other South Asian rivers, such as the Ganga River and Inner Asian rivers. It is suspected discharge data in many of these rivers are greatly affected by similar errors of flow measurements. The hydrologic studies following the IPCC may err too much on the side of caution when evaluating “errors” related to the so called ‘Karakoram Anomaly’, which affects both the UIR and YKR. No one wants to make a mistake such as the exaggerated report for Himalayan glacier retreat^17^. However, in an attempt to avoid these types of errors, it is important not to err on the opposite side. We urge scientists to accurately report the full range of possible outcomes, even if they are improbable, controversial or poorly understood.

## Materials and Methods

The available dataset in this study is consist of daily and monthly river discharge data from 5 and 8 stations on the major tributaries and the upstream and main stem of the Indus and 1 gauge LG of the YKR is in the China-KK. These data were obtained from the Hydrology Bureau of Xinjiang of China, the WAPDA of Pakistan and the Dumkar-NHPC of India. Monthly averages were calculated from daily records. In addition, monthly mean precipitation records for various periods at certain valley-based stations were obtained from the Meteorology Department of Pakistan (PMD). The study area above the KC station covers an area of 174,073 km^2^ that is sandwiched between the E-W-trending HKT. The studied Indus consists of 5 large inflows from the SGR, SYR, the KM upstream and the main stew KC, then the river Jhelum (JLR) and Chenab upstream (CNR). Their hydrometric stations are at Mangla dam of the Jhelum and Marala dam of the Chenab, and at KC, 12 km east of SG, 42 km eastward to SYR (Yugo), 110 km to the KM in the upper Indus, and 146 km from Dumkar in India. Their climate station is at Skardu in the Pak-Kashmir and Leh in the India-Kashmir. According to the same pattern of both the regional climate and surface flow on the hydrology in the KK rivers, we explored the teleconnections of monthly flow in the summer melt period (Jul.-Sep.) among the YKR, SGR, SYR and KC. For 38 years (see SI2) a close similarity was shown in meltwater flow between the YKR and the SYR (0.24–0.7, p < 0.01) and reliable correlations between the YKR and the KC (0.16–0.52, p < 0.05). Both close and poor correlations between the YKR and the SGR were found for 12 years (0.85, 0.54; p < 0.05, 0.21, 0.09). There was no correlation between the YKR and the KM in Jun. and Jul. (0.26, 0.50; p < 0.05), whereas it was favorable in Aug. and Sep. for 22 years. It is suggested that the poor correlations may not be real, but a result of unreliable flow measurements or estimates of the UIR. More reliable data can be applied to partially correct the poor monthly data. It shows the positive and negative biases occurred before and after the 1990s, primarily from Jun. to Aug., coinciding with poor correlation between the T-Q curves among the river flows. The hydrologic and snow-ice parameters of the rivers are listed in Table 1.

The water budget: at monthly and annual scales of the upper Indus should be in the following balance:

1 $${{\rm{Q}}}_{{\rm{kc}}}={{\rm{Q}}}_{{\rm{sgr}}}+{{\rm{Q}}}_{{\rm{syr}}}+{{\rm{Q}}}_{{\rm{km}}}+{{\rm{Q}}}_{{\rm{ungauge}}}$$

The hydrologic theory of a rationality test between or among these adjacent rivers at daily and monthly time steps is as follows: (1) The flow of a small watershed (glacier/snow area) must be smaller than that of the large watershed; otherwise, the flow of the small watershed is an error; (2) daily anomalies of flow and the extreme flood must have a reasonable temperature or rainfall; otherwise, they are in error; (3) the flow of the KM (down- stream) must be greater than that of the DK (upstream); otherwise, there is an error in the DK; (4) the allowed thresholds of daily and monthly anomalies are ±15% and ±5% in the melting period and ±5% and ±2.5% in the winter, respectively.

### Correlation analysis

Temperature index or snow melt models rest upon a claimed relationship between snow-ice meltwater and air temperature usually expressed in the form of positive temperatures^8,9^. As air temperature generally is the most readily available data type, such models are the most widely used methods in snow-ice melt computations for many purposes, such as hydrological modeling, ice dynamic modeling and climate sensitivity studies. The analogue method assumes that if two hydrological regimes are similar regarding the large-scale hydrological climatic cycle, they should also be similar with respect to the sub-basin conditions. Local hydrological conditions depend on the synoptic situation, but local features, such as orography and surface properties, also play an important role^40^.

## Electronic supplementary material

Supporting information

## Data Availability

The data sets of local observations (air temperature, streamflow) are available from PMD and WAPDA but restrictions apply to the availability of these data, which are used under license for this study, and so are not publicly available. The daily data are however available from the authors upon reasonable request and with permission of PMD and/or WAPDA. Estimated data of the glaciated area in both the Indus subbasin and in the YKR of China is based on the Randolph Glacier Inventory (version 5.0) and the Glacier Inventory of China (version 2.0), respectively.
