# Supplementary material for: Large observational bias on discharge in the Indus River since 1970s
Source: Sci Rep. 2018 Nov 23;8:17291. doi: 10.1038/s41598-018-35600-3 (PMC6251862; doi:10.1038/s41598-018-35600-3)
Supplement: Supplementary file 1 — Supporting information [file 41598_2018_35600_MOESM1_ESM.pdf]

Another montage on discharge in the Indus River

Jingshi Liu[1], Shichang Kang[1,2.3], Kenneth Hewitt[4], Linjin Hu[5], Li Xianyu[6]

1. Key Laboratory of Tibetan Environment Changes and Land Surface Processes, Institute of Tibetan Plateau Research, Chinese Academy of Sciences, Beijing, China；[2]State key laboratory of cryospheric sciences, Northwest Institute of Eco-environment and Resources, Chinese Academy of Sciences(CAS), Lanzhou; [3]CAS Center for Excellence in Tibetan Plateau Earth Sciences, Beijing, China；[4]Cold region research center, Wilfird Laurier University, Waterloo, Ontario, Canada；[5]Hydrology and water resources bureau of Xinjiang, Urumqi, Xinjiang, China; [6]Hydrology and water resources bureau of the Yangtze River, Wuhan, China

Correspondence and requests for materials should be addressed to J.S.Liu (email: jsliu@itpcas.ac.cn)

Supplementary Information

SI1 Original discharge data from published paper

Table Monthly flow contribution from subbasins to the main stew at Kachura in the UIR

	Khamong %	Shyok %	Shigar %	Kachura %
Jan.	50.78	21.13	13.64	91.54
Feb.	52.31	26.91	14.8	94.02
Mar.	57.81	23.4	14.98	96.19
Apr.	61.07	17.51	12.31	90.88
May	56.77	14.84	8.92	80.53
Jun.	52.41	22.87	14.86	90.14
Jul.	37.9	38.38	21.88	98.14
Aug.	32.76	42.51	21.94	97.21
Sep.	36.68	36.32	23.21	97.2
Oct.	44.99	32.07	14.85	91.91
Nov.	47.15	29.56	13.32	90.04
Dec.	50.23	27.72	12.37	90.32

From Biswajit W. A quantitative assessment of the genetic sources of the hydrologic flow regimes in Upper Indus Basin and its significance in a changing climate, J. Hydrol., 509, 549–572 (2014).

SI2 the telecorrelation between the studied stations

Telecorrelations between monthly discharges

	YKR-SGR	YKR-SYR	YKR-KC	SGR-SYR	SYR-KC
Jun	**0.85**	0.23	0.16	0.66	0.48
Jul	0.54	0.57	0.52	**0.86**	0.52
Aug	0.21	0.70	0.43	0.59	0.66
Sep	0.09	0.54	0.44	0.15	0.74

Although they are closely adjacent rivers and are ice snow fed with high correlation in air temperature, the teletcorrelaltion are extreme variable with the lowest 0.09 and highest 0.86, the highest correlation does not occur in July when the highest correlation of the temperature occur. The lowest correlation 0.15 in Sep. even occurs in SGR-SYR. These irrationals can be evidenced by the cross test in SI3 below.

YKR,SGR,SYR and KC has monthly discharge data 1961-2012;1985-92,94,96-98;1973-2011 except for 1975/2009 and 1982-2009, respectively.

SI3 daily and monthly data for the rational test

The daily river stage and discharge at Jhelum (JLR) and Chenab River (CNR)

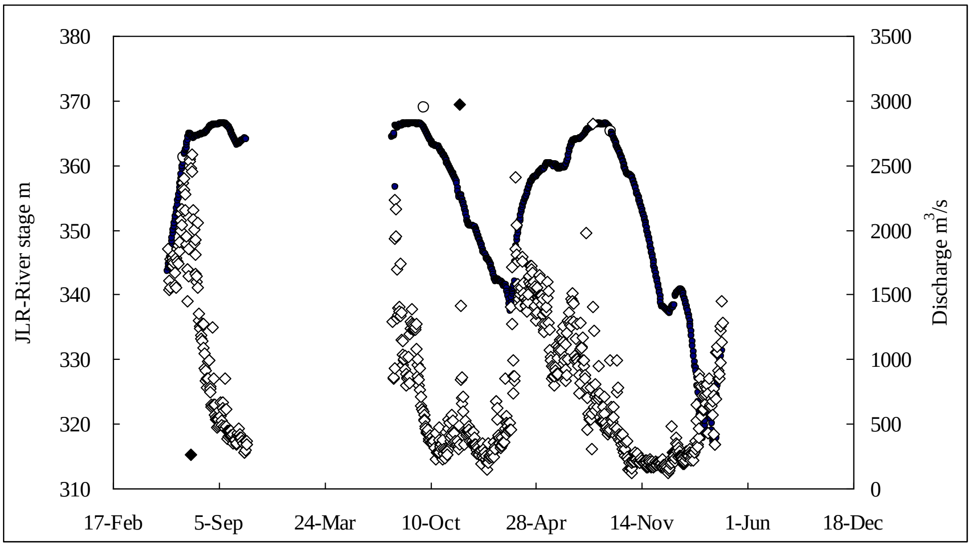

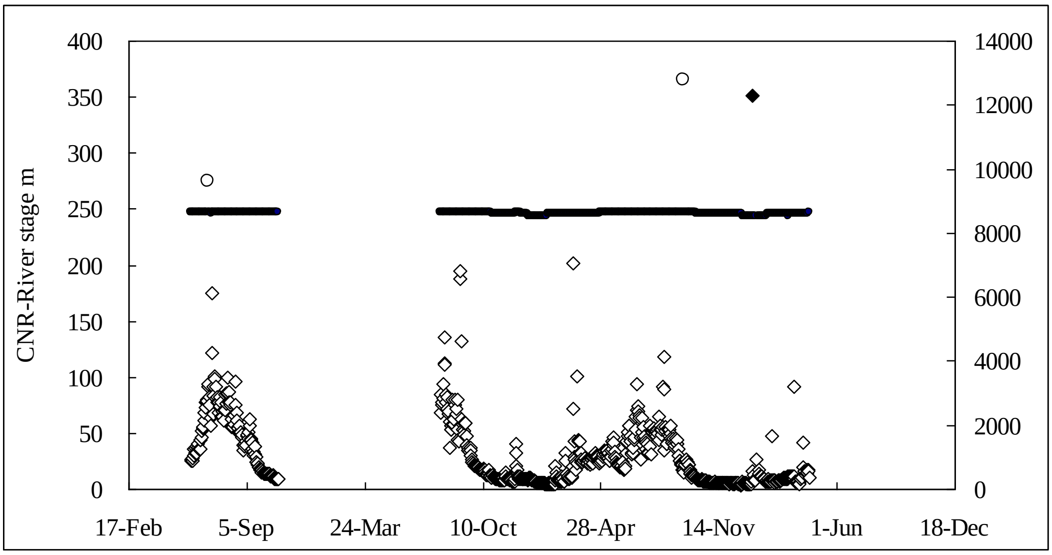

SI3-Fig. 1 Hydrographs of daily river stage and discharge in Jhelum and Chenab River in Jun. – Oct. 2005 and from Aug. 2006 to Apr. 2008. The ● and ○ represent the river stage and its extremes, the ◇ and ◆ represent the discharge and its extremes, respectively. It is clear the negative curves between the daily stage and discharge when the stage were upward the discharge were decline, all extreme (the highest and lowest) stages were not responsible to the extreme (maximum and minimum) discharge. The daily stage dropped to the lowest 317 m from 343 m in Jhelum, as the same, the stage jumped to the highest 365 m from 247 m in the Chenab. The daily discharge jumped to the maximum 2974 m^3/s from 340 m^3/s in Dec. 2006 in the Jhelum, the discharge jumped to the maximum 12288 m^3/s from 246 m^3/s in Jan. 2008 in the Chenab.

The data from the online records of Pakistan Meteorology Department from 2005 to 2008.

SI3-table 1 The daily data marked in yellow is irrational by the cross test

YY	MM	DD	Shigar	Shyok	YY	MM	DD	Shigar	Shyok
1985	5	12	58.3	61.4	1986	5	13	**55.5**	54.1
1985	5	13	61.7	62.9	1986	5	14	**61.7**	54.6
1985	5	14	65.1	58.9	1986	5	15	**62.3**	60.0
1985	5	15	69.6	59.7	1986	5	16	**68.8**	71.6
1985	5	16	71.6	60.0	1986	5	17	**72.2**	73.6
1985	5	17	73.0	55.2	1986	5	18	**81.5**	74.5
1985	5	18	77.3	52.7	1986	5	19	**87.8**	68.8
1985	5	19	80.1	52.4	1986	5	20	**90.0**	62.9
1985	5	20	80.7	57.2	1986	5	21	**108.2**	61.7
1985	5	21	83.0	63.7	1986	5	22	**101.4**	58.6
1985	5	22	87.2	69.4	1986	5	23	**94.6**	56.6

1985	5	23	92.0	84.1
1985	5	24	97.1	110.7
1985	5	25	**103.9**	101.6
1985	5	26	**109.9**	110.7
1985	5	27	**112.4**	102.2
1985	5	28	**117.2**	105.0
1985	5	29	**122.3**	129.1
1985	5	30	**127.1**	138.2
1985	5	31	**132.2**	131.4
1985	6	01	**137.0**	112.4
1985	6	02	**145.0**	124.0
1985	6	03	**150.1**	120.9
1985	6	04	**160.8**	139.9
1985	6	05	167.6	182.3
1985	6	06	172.1	250.6
1985	6	07	180.1	311.4
1985	6	08	183.5	129.1
1985	6	09	186.9	169.9
1985	6	10	219.7	191.4
1985	6	11	224.8	300.1
1985	6	12	200.5	319.9
1985	6	13	195.9	191.4
1985	6	14	200.5	322.8

? Qsgr > Qsyr

1986	5	24	**107.3**	56.1
1986	5	25	**93.7**	56.1
1986	5	26	**102.2**	58.3
1986	5	27	**88.6**	58.3
1986	5	28	**83.0**	57.8
1986	5	29	**80.7**	55.8
1986	5	30	**73.6**	57.2
1986	5	31	**83.5**	62.6
1986	6	01	**83.0**	59.7
1986	6	02	**92.9**	58.3
1986	6	03	**105.6**	58.3
1986	6	04	**120.3**	60.3
1986	6	05	**139.0**	59.7
1986	6	06	**156.3**	59.5
1986	6	07	**175.5**	66.0
1986	6	08	**177.8**	81.0
1986	6	09	**173.3**	99.1
1986	6	10	**167.6**	145.8
1986	6	11	**167.6**	78.1
1986	6	12	**157.4**	144.7
1986	6	13	**137.0**	139.9
1986	6	14	**123.2**	58.0
1986	6	15	**136.2**	113.8
1986	6	16	**145.0**	139.0
1986	6	17	**143.0**	133.1
1986	6	18	152.0	143.3
1986	6	19	158.6	220.0
1986	6	20	171.0	288.8
1986	6	21	178.9	396.4
1986	6	22	193.7	472.8
1986	6	23	236.1	628.5
1986	6	24	202.7	690.8
1986	6	25	212.9	727.6
1986	6	26	242.6	702.2
1986	6	27	297.3	560.6
1986	6	28	334.1	506.8
1986	6	29	373.7	453.0
1986	6	30	328.4	430.4
1986	7	01	297.3	478.5
1986	7	02	319.9	396.4
1986	7	03	359.6	339.8
1986	7	04	407.7	421.9
1986	7	05	455.8	458.7
1986	7	06	484.1	651.2
1986	7	07	487.0	923.0
1986	7	08	438.8	1073.0
1986	7	09	390.7	1146.7
1986	7	10	339.8	1024.9
1986	7	11	288.8	968.3
1986	7	12	277.5	917.3
1986	7	13	241.2	812.6

? Qsgr > Qsyr

YY	MM	DD	Shigar	Shyok
1988	5	17	96.8	115.8
1988	5	18	109.6	126.8
1988	5	19	**121.5**	114.7
1988	5	20	**129.4**	115.5
1988	5	21	**141.6**	139.0
1988	5	22	**152.9**	141.3
1988	5	23	**172.1**	122.3
1988	5	24	**190.3**	134.8
1988	5	25	**204.1**	135.9
1988	5	26	**216.6**	143.3
1988	5	27	**233.6**	153.5
1988	5	28	**229.3**	157.4
1988	5	29	**232.2**	175.0
1988	5	30	**243.5**	192.5
1988	5	31	229.3	252.8

? Qsgr > Qsyr

1990	6	11	262.7	331.6
1990	6	12	330.8	320.9
1990	6	13	**447.6**	339.4
1990	6	14	**497.6**	333.1
1990	6	15	**507.3**	335.0
1990	6	16	**541.7**	339.3
1990	6	17	**589.9**	354.3
1990	6	18	**665.9**	398.5
1990	6	19	**714.3**	523.7
1990	6	20	**703.2**	645.2
1990	6	21	**756.6**	684.9
1990	6	22	768.2	901.5
1990	6	23	964.9	1226.0
1990	6	24	1038.0	1378.0
1990	6	25	1059.0	1448.0
1990	6	26	1082.0	1453.0
1990	6	27	1018.0	1327.0
1990	6	28	981.0	1303.0
1990	6	29	974.2	1414.0
1990	6	30	1010.0	1600.0
1990	7	01	1014.0	1747.0
1990	7	02	991.4	1752.0
1990	7	03	965.6	1858.0
1990	7	04	1004.0	1889.0
1990	7	05	1198.0	1898.0
1990	7	06	992.5	1785.0
1990	7	07	962.5	1775.0
1990	7	08	1176.0	1890.0
1990	7	09	1434.0	**2001.0**
1990	7	10	1241.0	**2037.0**
1990	7	11	1051.0	1784.0
1990	7	12	733.8	1461.0
1990	7	13	592.8	1220.0
1990	7	14	661.8	1132.0
1990	7	15	909.3	1109.0
1990	7	16	965.6	1205.0
1990	7	17	1032.0	1220.0
1990	7	18	1068.0	1155.0
1990	7	19	1073.0	1087.0
1990	7	20	**1031.0**	**1023.0**
1990	7	21	**1020.0**	**967.9**
1990	7	22	884.8	987.9

?Qsgr > Qsyr in Jun12-21/Jul.20-21.

YY	MM	DD	Shigar	Shyok
1988	11	10	39.1	103.9
1988	11	11	39.1	102.8
1988	11	12	42.5	102.2
1988	11	13	30.0	102.2
1988	11	14	31.1	101.6
1988	11	15	28.9	101.4
1988	11	16	36.8	100.5
1988	11	17	35.1	99.9
1988	11	18	37.9	98.5

1990	9	15	513.0	695.8
1990	9	16	526.9	719.5
1990	9	17	580.3	708.5
1990	9	18	488.5	696.7
1990	9	19	395.1	713.9
1990	9	20	292.0	699.1
1990	9	21	239.3	652.3
1990	9	22	87.4	621.8
1990	9	23	60.4	478.0
1990	9	24	34.8	427.7

1988	11	19	39.1	96.5
1988	11	20	39.1	96.3
1988	11	21	43.6	95.1
1988	11	22	56.9	94.8
1988	11	23	49.3	94.6

Q increased in SGR in winter? but decreased in SYR.

?Qsgr suddenly dropped on 22th .

1988	8	11	702.2	1361.8
1988	8	12	747.5	1353.3
1988	8	13	**1489.2**	1429.8
1988	8	14	979.6	1511.9

? Qsgr a jumped to max flood? And suddenly > Qsyr ?

1991	4	04	37.1	48.3
1991	4	05	41.2	48.4
1991	4	06	47.3	47.5
1991	4	07	52.0	47.3
1991	4	08	50.8	44.9
1991	4	09	50.6	47.7
1991	4	10	49.0	47.1
1991	4	11	49.2	44.9
1991	4	12	48.7	45.0
1991	4	13	45.2	45.9
1991	4	14	48.1	56.2

? Qsgr > Qsyr

1991	5	29	99.3	112.7
1991	5	30	109.5	107.1
1991	5	31	118.8	109.4
1991	6	01	118.6	120.3
1991	6	02	122.6	162.6

? Qsgr > Qsyr

YY	MM	DD	Shigar	Shyok
1991	9	26	155.3	327.6
1991	9	27	160.3	316.5
1991	9	28	143.3	291.0
1991	9	29	123.8	267.7
1991	9	30	83.0	248.7
1991	10	01	53.8	225.2
1991	10	02	43.4	205.3
1991	10	03	47.7	191.5
1991	10	04	47.4	180.8
1991	10	05	44.4	168.7

Qsgr suddenly dropped, but not in Qsyr

YY	MM	DD	Shigar	Shyok
1992	4	14	29.7	36.7
1992	4	15	37.4	37.0
1992	4	16	43.0	37.5
1992	4	17	50.1	37.5
1992	4	18	55.4	37.6
1992	4	19	56.6	37.4
1992	4	20	57.8	37.3
1992	4	21	54.7	37.4
1992	4	22	54.7	37.5
1992	4	23	56.0	37.3
1992	4	24	55.8	38.0
1992	4	25	44.3	39.6
1992	4	26	46.4	41.0
1992	4	27	23.4	41.7
1992	4	28	34.5	41.9
1992	4	29	112.5	44.7
1992	4	30	116.7	44.4
1992	5	01	91.3	44.4

1992	5	02	90.5	44.9
1992	5	03	115.2	43.8
1992	5	04	98.1	43.8
1992	5	05	88.3	42.8
1992	5	06	91.0	42.2
1992	5	07	90.9	41.3
1992	5	08	90.9	40.9
1992	5	09	99.2	40.4
1992	5	10	101.9	40.7
1992	5	11	121.9	42.7
1992	5	12	142.0	49.8
1992	5	13	131.5	91.5
1992	5	14	120.3	85.3
1992	5	15	120.2	86.9
1992	5	16	125.4	89.5
1992	5	17	115.9	102.3
1992	5	18	96.7	94.9
1992	5	19	90.3	95.5
1992	5	20	80.6	88.8
1992	5	21	105.5	86.9
1992	5	22	105.7	87.5
1992	5	23	137.6	106.4
1992	5	24	137.3	113.9
1992	5	25	145.2	117.8
1992	5	26	165.0	156.9
1992	5	27	161.9	125.3
1992	5	28	153.3	117.0
1992	5	29	168.8	115.8
1992	5	30	167.2	107.2
1992	5	31	171.0	105.3
1992	6	01	177.3	110.9
1992	6	02	186.1	130.3
1992	6	03	203.1	161.8
1992	6	04	201.4	162.4
1992	6	05	208.7	226.7

? Qsgr > Qsyr

YY	MM	DD	Shigar	Shyok
1991	12	22	15.3	69.7
1991	12	23	14.6	68.3
1991	12	24	7.4	62.5
1991	12	25	6.3	62.3
1991	12	26	7.0	61.8
1991	12	27	5.5	61.7
1991	12	28	11.7	64.2
1991	12	29	11.1	62.4
1991	12	30	11.2	59.7
1991	12	31	11.4	65.5
1992	1	01	11.9	65.0
1992	1	02	15.2	59.4
1992	1	03	16.6	58.4
1992	1	04	18.0	56.4
1992	1	05	16.4	57.4
1992	1	06	14.6	56.7
1992	1	07	10.3	50.5
1992	1	08	10.7	53.7
1992	1	09	12.4	54.6
1992	1	10	18.5	54.1
1992	1	11	16.1	53.6
1992	1	12	19.9	52.2
1992	1	13	24.7	52.5
1992	1	14	29.0	52.1
1992	1	15	38.5	53.2
1992	1	16	43.6	53.5
1992	1	17	18.2	53.2

1992	1	18	11.1	51.9
1992	1	19	24.1	51.8
1992	1	20	30.7	51.5
1992	1	21	33.1	52.8
1992	1	22	32.8	51.2
1992	1	23	32.5	50.7
1992	1	24	35.5	53.2
1992	1	25	28.2	55.3
1992	1	26	28.9	55.2
1992	1	27	28.7	56.1
1992	1	28	26.4	54.9
1992	1	29	25.6	54.1
1992	1	30	28.4	53.5
1992	1	31	30.7	53.1
1992	2	01	31.6	52.3

Qsgr suddenly dropped/jumped in cold Jan., but in SYR
Qsgr drop and jump to the historical minima 5.5 in the 12 yrs

1992	9	17	182.9	286.8
1992	9	18	160.0	263.8
1992	9	19	145.1	580.9
1992	9	20	140.0	231.4
1992	9	21	143.4	219.2

? Qsyr suddenly jumped

1996	3	23	39.2	42.04
1996	3	24	41.3	42.15
1996	3	25	42.5	42.41
1996	3	26	43.1	42.33
1996	3	27	44.8	42.75
1996	3	28	45.2	42.07
1996	3	29	44.6	46.19
1996	3	30	43.5	45.67
1996	3	31	43.1	43.73
1996	4	01	42.2	43.41
1996	4	02	42.4	42.64
1996	4	03	42.7	42.12
1996	4	04	41.8	41.94
1996	4	05	41.5	42.97

? Qsgr < > Qsyr

YY	MM	DD	Shigar	Shyok
1996	5	11	53.7	60.99
1996	5	12	55.6	58.95
1996	5	13	59.1	55.72
1996	5	14	63.1	52.98
1996	5	15	65.4	54.07
1996	5	16	68.0	52.66
1996	5	17	67.6	53.01
1996	5	18	67.6	52.6
1996	5	19	167.7	52.38
1996	5	20	195.3	57.19
1996	5	21	200.7	61.56
1996	5	22	156.0	62.94
1996	5	23	64.9	61.9
1996	5	24	64.6	62.55
1996	5	25	141.8	62.45
1996	5	26	151.6	80.6
1996	5	27	177.0	109.4
1996	5	28	151.9	141.4
1996	5	29	129.0	152.1

? Qsgr suddenly drop/jump and ? Qsgr > Qsyr

1996	9	28	361.9	463.3
1996	9	29	330.1	427
1996	9	30	306.1	412.7
1996	10	01	289.4	406.3
1996	10	02	305.0	390.9

1996	10	03	240.5	363.9
1996	10	04	65.0	324.8
1996	10	05	56.2	275.5
1996	10	06	51.6	266.1
1996	10	07	47.1	230.7

Qsgr suddenly drop, but Qsyr

YY	MM	DD	Shigar	Shyok	YY	MM	DD	Shigar	Shyok
1994	5	13	65.5	57.1	1997	5	04	88.5	75.12
1994	5	14	75.2	57.6	1997	5	05	81.9	72.15
1994	5	15	93.0	58.6	1997	5	06	73.0	76.05
1994	5	16	98.1	76.4	1997	5	07	76.3	95.37
1994	5	17	73.0	74.0	1997	5	08	45.1	90.3
1994	5	18	98.7	100.3	1997	5	09	33.8	88.93
1994	5	19	100.0	116.5	1997	5	10	52.6	84.2
1994	5	20	103.8	117.3	1997	5	11	85.5	79.87
1994	5	21	**121.6**	118.0	1997	5	12	58.1	77.97
1994	5	22	**148.6**	122.0	1997	5	13	33.7	73.76
1994	5	23	**148.9**	130.9	1997	5	14	35.5	71.34
1994	5	24	**149.2**	131.3	1997	5	15	36.5	69.4
1994	5	25	**155.3**	135.7	1997	5	16	54.0	65.32
1994	5	26	**170.8**	133.4	1997	5	17	90.7	66.26
1994	5	27	**205.0**	145.6	1997	5	18	77.0	65.99
1994	5	28	**244.7**	166.9	1997	5	19	82.6	64.33
1994	5	29	**257.7**	182.8	1997	5	20	111.9	65.23
1994	5	30	**279.0**	203.0	1997	5	21	127.1	65.39
1994	5	31	**262.4**	217.1	1997	5	22	130.1	68.59
1994	6	01	**256.1**	193.4	1997	5	23	148.3	75.05
1994	6	02	**314.5**	180.8	1997	5	24	140.5	78.91
1994	6	03	**315.9**	181.7	1997	5	25	117.3	90.1
1994	6	04	**293.7**	198.6	1997	5	26	175.7	104.7
1994	6	05	**273.7**	194.8	1997	5	27	200.5	126.1
1994	6	06	**332.4**	215.3	1997	5	28	219.4	124.3
1994	6	07	**336.6**	254.7	1997	5	29	225.0	126.1
1994	6	08	**368.1**	320.8	1997	5	30	235.2	138.5
1994	6	09	**350.6**	270.4	1997	5	31	222.7	150.3
1994	6	10	**358.3**	227.8	1997	6	01	251.8	159.4
1994	6	11	**365.0**	207.9	1997	6	02	251.1	177.6
1994	6	12	**355.2**	202.4	1997	6	03	267.8	197.9
1994	6	13	**369.9**	202.5	1997	6	04	286.4	219.2
1994	6	14	**402.0**	190.0	1997	6	05	292.0	235.2
1994	6	15	**401.8**	183.7	1997	6	06	314.3	245.6
1994	6	16	**423.0**	184.8	1997	6	07	318.0	239.6
1994	6	17	**424.1**	200.4	1997	6	08	325.3	244.4
1994	6	18	**387.7**	238.8	1997	6	09	369.9	269.5
1994	6	19	**350.7**	292.7	1997	6	10	358.2	269.8
1994	6	20	345.0	376.8	1997	6	11	344.2	274.6
					1997	6	12	332.3	279.7
					1997	6	13	272.2	267.7
					1997	6	14	217.7	259.1
					1997	6	15	177.9	258.8

? Qsgr > Qsyr   ? Qsgr > Qsyr

YY	MM	DD	Shigar	Shyok	YY	MM	DD	Shigar	Shyok
1997	6	26	606.8	768.4	1997	9	24	166.9	496.6
1997	6	27	697.1	841.7	1997	9	25	162.9	441.2
1997	6	28	713.9	832.9	1997	9	26	180.9	407
1997	6	29	532.9	773.8	1997	9	27	176.4	369.9
1997	6	30	405.5	732.5	1997	9	28	211.1	369.8
1997	7	01	193.2	551.8	1997	9	29	213.1	360.8
1997	7	02	64.4	439.9	1997	9	30	172.1	352.7
1997	7	03	41.2	391.1	1997	10	01	60.4	333.8
1997	7	04	41.2	378.3	1997	10	02	49.3	317.7
1997	7	05	48.3	443.4	1997	10	03	41.3	308.4
1997	7	06	227.6	551.1	1997	10	04	50.3	305.7

1997	7	07	349.8	687.1	1997	10	05	57.8	296.2
1997	7	08	511.8	849.4	1997	10	06	57.8	287.1
					1997	10	07	65.0	279.7
					1997	10	08	54.1	267
					1997	10	09	37.8	264.5

Qsgr suddenly drop/jump Qsgr suddenly drop, but not in Qsyr

1986	8	16	560.6	1189.1	1980	6	22	781.4
1986	8	17	628.5	1194.8	1980	6	23	849.4
1986	8	18	622.9	1149.5	1980	6	24	1101.4
1986	8	19	583.2	1208.9	1980	6	25	302.9
1986	8	20	574.7	**2010.2**	1980	6	26	283.1
1986	8	21	521.0	985.3	1980	6	27	1013.6
1986	8	22	444.5	789.9	1980	6	28	886.2
1986	8	23	424.7	705.0				

Qsyr suddenly jumped max flood? But None in Qsgr?/ ? Qsyr suddenly drop/jump/drop;

YY	MM	DD	Shyok
1976	12	25	**118.6**
1976	12	26	**117.8**
1976	12	27	**116.1**
1976	12	28	59.2
1976	12	29	58.9
1976	12	30	58.0
1976	12	31	56.6
1977	1	01	62.3
1977	1	02	62.9
1977	1	03	61.4
1977	1	04	62.3
1977	1	05	64.0
1977	1	06	62.3
1977	1	07	60.3
1977	1	08	58.6
1977	1	09	60.9
1977	1	10	66.0

YY	MM	DD	Shyok
1976	11	25	204.7
1976	11	26	205.0
1976	11	27	203.9
1976	11	28	77.6
1976	11	29	76.7
1976	11	30	76.4
1976	12	01	76.2
1976	12	02	74.7
1976	12	03	74.2
1976	12	04	**181.2**
1976	12	05	**173.3**
1976	12	06	**171.6**
1976	12	07	**163.1**

Qsyr suddenly drop in winter ?/ Qsyr suddenly drop/jump in winter ?

YY	MM	DD	Shyok
1980	12	27	48.1
1980	12	28	48.1
1980	12	29	48.1
1980	12	30	47.6
1980	12	31	46.1
1981	1	01	177.0
1981	1	02	175.8
1981	1	03	175.8
1981	1	04	175.0
1981	1	05	174.7
1981	1	06	173.0
1981	1	07	170.7
1981	1	08	169.9
1981	1	09	169.9
1981	1	10	168.2
1981	1	11	167.9
1981	1	12	166.8
1981	1	13	165.6
1981	1	14	163.6
1981	1	15	164.8
1981	1	16	163.9
1981	1	17	163.9
1981	1	18	159.1
1981	1	19	160.2
1981	1	20	161.9
1981	1	21	163.9

1981	1	22	162.8
1981	1	23	161.7
1981	1	24	161.1
1981	1	25	162.2
1981	1	26	160.8
1981	1	27	160.8
1981	1	28	163.1
1981	1	29	163.1
1981	1	30	160.8
1981	1	31	159.1
1981	2	01	158.0
1981	2	02	156.9
1981	2	03	155.2
1981	2	04	153.7
1981	2	05	154.0
1981	2	06	154.3
1981	2	07	153.7
1981	2	08	149.2
1981	2	09	145.0
1981	2	10	152.0
1981	2	11	156.9
1981	2	12	158.3
1981	2	13	160.8
1981	2	14	163.1
1981	2	15	163.9
1981	2	16	163.1
1981	2	17	160.8
1981	2	18	160.2
1981	2	19	160.8
1981	2	20	160.8
1981	2	21	160.0
1981	2	22	159.7
1981	2	23	159.7
1981	2	24	160.0
1981	2	25	160.0
1981	2	26	159.7
1981	2	27	159.7
1981	2	28	159.4
1981	3	01	144.1
1981	3	02	132.2

? Qsyr suddenly **jump from 46 to177 on the new year day ? resulted in the maximum monthly Q in Jan. – Mar.**

1986	6	06	**156.3**	59.5
1986	6	07	**175.5**	66.0
1986	6	08	**177.8**	81.0
1986	6	09	**173.3**	99.1
1986	6	10	**167.6**	145.8
1986	6	11	**167.6**	78.1
1986	6	12	**157.4**	144.7
1986	6	13	**137.0**	139.9
1986	6	14	**123.2**	58.0
1986	6	15	**136.2**	113.8
1986	6	16	**145.0**	139.0
1986	6	17	**143.0**	133.1

?Qsgr > Qsyr? Qsyr drop/jump in meltwater ? but Qsgr

			Q	T
2010	8	1	2089	20.85
2010	8	2	2035	23
2010	8	3	2280	24.4
2010	8	4	2656	25.05
2010	8	5	3059	22.55
2010	8	6	3812	21.95
2010	8	7	4119	20.9
2010	8	8	3696	22.65

2010	8	9	3294	18.8
2010	8	10	2953	21.8
2010	8	11	2653	22.7
2010	8	12	2534	23.55
2010	8	13	2472	23.65

? the annual Q on the 7th, but the highest T on the 4th; the lowest T on the 9th, but the high Q in KC

SI3-Fig. 2 Daily discharge vs air temperature in meltwater month

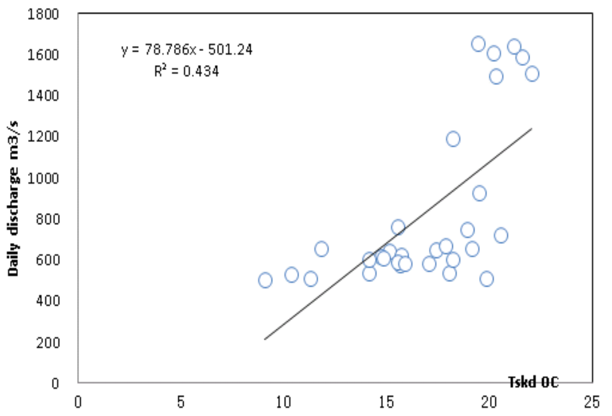

1993May

SGR : 6 big and small discharges in two groups ?

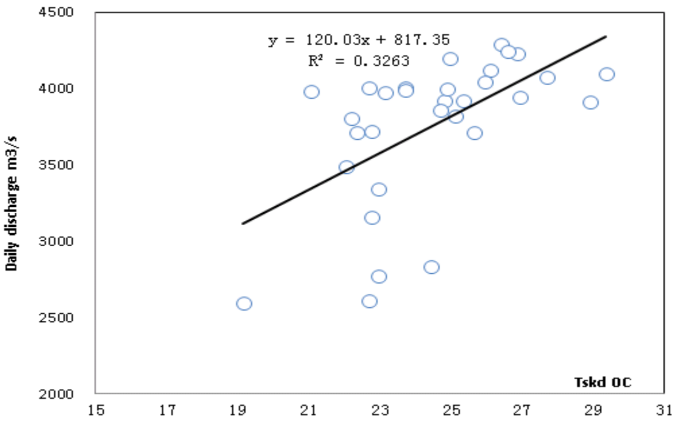

1998Jul

KC : 4 small discharges out of the up group?

SI3-Fig. 3 Kchura the Q-T daily point during Jun. to Aug.

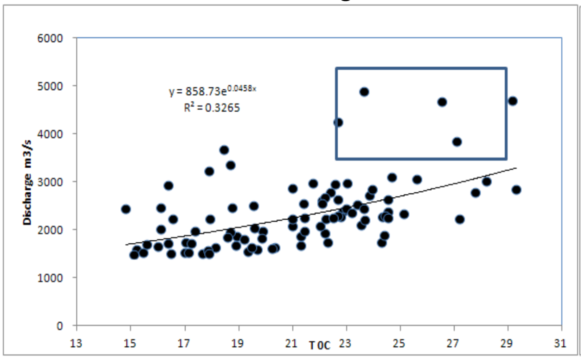

1989: bad

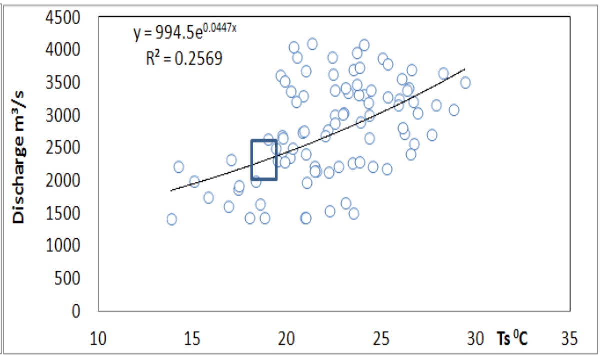

2004: biggest Q at near 21℃?

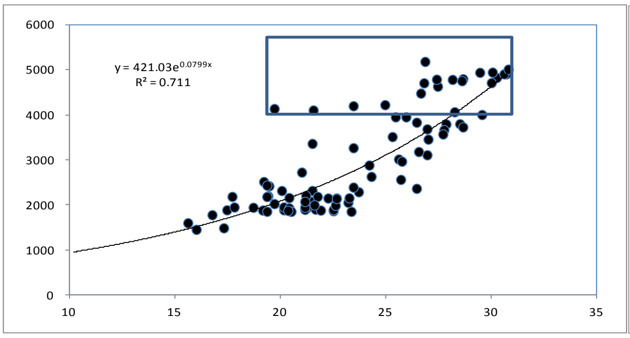

1982: 6 same flows at about 4100 in box, but T 20-30℃?

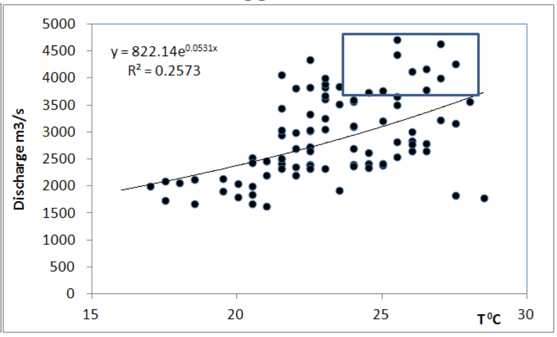

1980 : ?2 low flows at the highest T

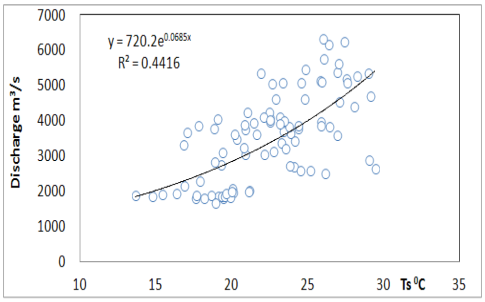

2006 : small Q at nearly highest 30℃?

SI3-table 2 Monthly discharge anomalies in Khamong

	Jan	Feb	Mar	Apr	May	Jun	Jul	Aug	Sep	Oct	Nov	Dec
1982	104.1	92.8	109.3	157.2	397.6	787.3	1044.9	1189.4	391.7	153.8	119.4	101.0
1983	97.5	87.2	98.0	132.8	418.4	1157.5	1511.2	1855.9	866.2	287.1	132.9	113.4
1984	109.9	99.3	107.7	157.2	501.6	1129.2	375.9	1206.8	537.5	174.8	119.2	95.2
1985	81.3	71.8	87.5	120.7	244.6	783.7	960.8	929.7	494.0	192.6	120.7	84.4
1986	74.9	78.6	90.3	130.7	327.8	1108.8	1468.3	1170.8	439.1	195.8	132.2	99.4
1987	80.2	75.0	97.3	123.7	239.7	977.3	1318.9	1000.6	506.4	221.5	110.9	114.9
1988	126.5	73.4	91.7	260.0	921.3	1543.6	1847.2	1221.6	518.6	270.5	170.2	130.4
1989	104.6	86.8	104.0	116.8	296.7	1158.3	1801.2	1186.7	484.6	220.3	132.8	123.3
1990	128.8	98.7	102.1	156.7	1297.8	1782.6	1570.6	1196.7	514.0	262.9	137.4	118.0
1991	105.5	92.8	114.1	174.0	383.3	1593.7	1694.2	1062.0	682.8	209.2	136.0	103.3
1992	94.7	78.0	91.4	123.9	397.7	1257.3	1439.2	1086.0	702.2	243.1	171.9	125.0
1993	100.7	85.4	93.3	153.6	599.0	1098.4	1220.2	748.2	475.0	216.8	141.2	119.0
1994	126.2	95.4	108.0	130.0	527.6	1138.2	1490.8	1429.2	615.0	216.2	161.7	146.9
1995	116.5	91.5	102.0	128.2	526.7	1040.8	1124.4	957.2	427.9	186.5	155.2	137.2
1996	114.7	96.4	114.0	205.0	369.3	1496.9	1438.3	1305.9	593.4	320.6	192.9	158.1
1997	144.3	114.0	136.2	144.5	405.4	735.8	1087.4	893.3	644.8	273.9	166.8	149.9
1998	106.5	86.3	106.5	170.7	503.8	1260.8	1842.9	1249.1	690.4	337.2	229.4	167.5
1999	144.5	129.5	144.9	319.9	1045.0	1319.9	1206.4	1134.2	751.4	416.0	164.6	128.1
2000	112.1	102.7	134.6	147.8	498.1	807.7	979.8	897.8	538.9	321.3	144.0	126.6
2001	113.5	102.2	119.6	125.1	299.7	708.0	792.3	559.9	388.5	180.6	129.5	122.2
2002	114.6	88.5	121.9	126.8	338.0	869.9	774.2	808.2	434.7	200.9	164.3	128.2
2003	104.0	105.1	125.6	157.5	633.5	1337.8	1410.9	853.5	409.5	269.8	163.1	131.8

Really could have much smaller discharge in July than that in Jun. and Aug. 1984 when the highest temperature in July ?
The summer monthly discharge became into small and small from Jun. to Aug. which temperature was higher and higher in 1990, 1996, 1999. = = =, and so on.

SI3-table 3 Discharge anomalies in Dumkar of the India-Kashmir

	Jan	Jan	Feb	Mar	Apr	May	Jun	Jul	Aug	Sep	Oct	Nov	Dec
1977	50.8	50.8	53.9	54.4	57.8	73.3	211.1	767.7	767.7	312.7	97.4	73.7	57.8
1978	47.4	47.4	51.6	54.4	69.6	238.7	518.3	759.7	864.9	285.3	94.5	69.6	57.7
1979	48.5	48.5	50.1	55.4	108.4	219.7	281.4	698.0	752.5	287.8	169.5	88.7	67.6
1980	52.0	52.0	49.4	59.0	66.4	161.9	320.9	740.3	771.4	168.6	80.2	67.7	57.0
1981	49.7	49.7	48.8	53.9	68.8	157.9	408.7	683.4				68.1	54.8
1982	49.2	49.2	48.7	53.4	64.4	136.9	324.9	743.0	945.9	235.6	93.3	70.6	58.8
1983	50.3	50.3	50.7	55.1	62.4	107.2	379.0	787.6	965.3	485.3	159.3	76.2	59.4
1984	48.8	48.8	48.6	53.9	65.7	97.1	433.5	461.6	785.3	227.4	84.2	69.6	58.5
1985	50.2	50.2	48.6	52.3	60.9	77.9	196.2	436.1	848.0	418.8	65.2	63.5	58.2
1986	49.2	49.2	48.0	50.5	58.9	73.3	296.9	702.0	498.9	320.4	90.6	67.8	55.4
1987	50.0	50.0	49.0	50.4	51.8	69.3	232.3	474.0	430.1	327.8	81.8	57.8	48.0
1988		27.0	20.7	23.0	49.4	127.9	353.4	828.2	875.2	261.4	89.8	63.6	61.2
1989	50.6	50.6	48.8	51.1	63.1	79.1	370.7	433.8	341.0	243.4	163.3	65.4	60.5
1990	54.4	54.4	48.4	50.4	60.8	185.3	547.8	473.4	612.2	473.2	193.5	102.7	78.1
1991	54.2	54.2	48.4	50.7	61.2	87.5	198.7	439.1	463.1	213.3	91.2	69.9	61.7
1992	52.6	52.6	47.9	48.2	53.5	152.8	512.9	368.1	498.5			68.3	60.8
1993	51.3	51.3	48.4			81.0	180.2	398.4	652.3	409.4	184.0	78.8	68.2
1994	52.1	52.1	48.7	48.5	51.6	69.6	138.8	556.8	787.7	531.9	195.1	66.7	61.5
1995	53.7	53.7	47.5	52.6	52.5	64.1					263.8	79.0	59.2
1996													
1997											177.7	70.8	52.8
1998	48.8	48.8	47.6	47.8	53.0	69.8	137.5						
1999					51.9	73.8	117.7	386.7					
2000												116.0	56.9
2001	47.7	47.7	47.7	47.7	50.8			647.6	565.3	236.8	124.4	142.6	96.3
2002	54.9	54.9	50.5	58.9	69.2	410.2	670.4	875.0	838.1	329.5	93.5	64.6	76.7
2003	49.0	49.0	65.0	84.8	120.3	332.9							

Really could have the same 54.4 in 3 March? Really could have many the same 47.7 in 2001, and many 48 in 1998?
Really could have much smaller discharge in July than that in Jun. and Aug. when the highest temperature in July in 1990 and 1992 ? = = =, and so on.

SI4-Fig. 1 Daily air temperature (T) to the discharge for YKR

Perfect correlations between T and Q in YKR

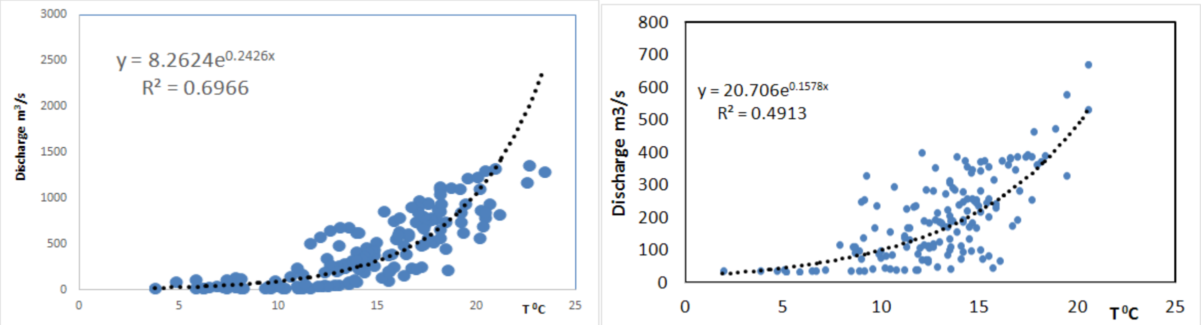

Perfect correlations in YKR: the high water in 1973; the low water in 1993

SI4-Fig. 2 Daily and monthly temperature to the discharge for SGR

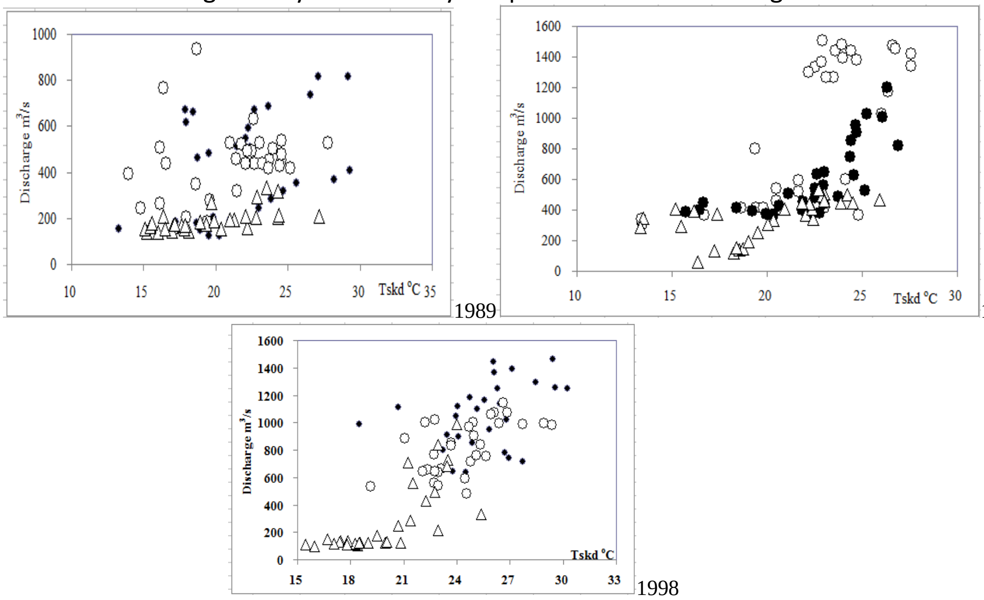

Fig. 2d Points of discharge correlated to air temperature in 1989, 1996 and 1998 for Shigar station (△-Jun,●-Jul,○-Aug), the correlation with a lag of 1 d illustrated. The ● and ○ points of Jul. and Aug. distribute in either large two-limbs or more random without a correlation representing the high flow year, the larger a flood, the larger the black points distribute. The maximum flood in 1996 and 1998 for example, the T risen from 22℃ to 28℃, the discharge parallel scattering without increase in Aug., and daily discharge jumped much bigger than that at 26℃ to 29℃. The points (△) in June mostly stand in either a two-limbs or in parallel. The random and two-limbs in daily correlations occurred in 8, 4,5, 3 and 5 times during May to Sep. from daily data of 12 yr.

SI4-Fig. 3 Monthly temperature to the discharge for Khamong and Dunkar

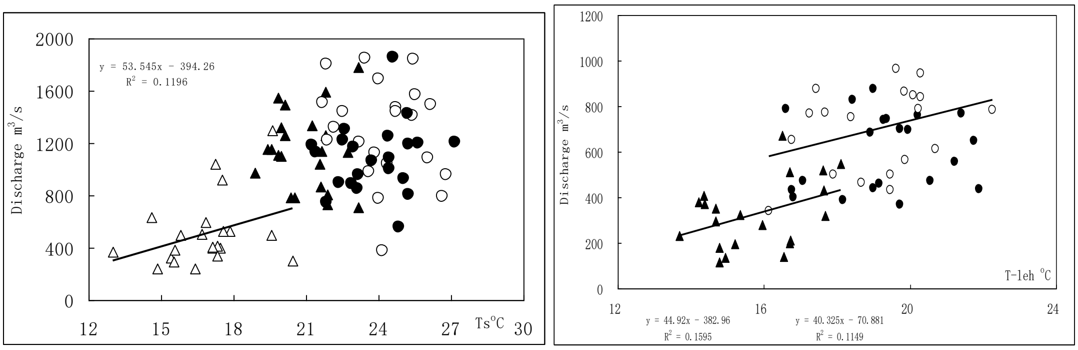

Fig. 2e Monthly correlation between air temperature and discharge during May to Sep at the KM upstream (left graph: 22 yr, R=0.33,P<0.1) and DK upstream (right graph: 21 yr, R=0.33,P<0.1); △-May,▲-Jun, ●-Jul, ○-Aug,◇-Sep.

SI5 Test of the annual floods

The annual extreme flood in the KK, especially the outburst one, is very dangerous and difficult to be directly observed, only the river stage can be captured sometimes if lucky. The meltwater flood of glaciers usually occur following the warmest weather in Jul. or Aug., and meet storm rain flood sometimes, there should be a consistency on occurring date. With data of annual daily flood during 1985 to 1998 at SG, SY and KC, both the flood at SG and KC in 1986 occurred on Aug. 3rd and 5th, but on the 20th at SY which strangely jumped to 2010 from 1210 on the 19th when a lower temperature than that in early Aug. As the same, the floods at both SY and KC in 1987 were on Jul. 25th, but on Aug. 24th at SG. The same cases occurred in 1990-92, 1994 and 1998 respectively. With the data during 1985 to 2008 at SY and KC, the earliest flood occurred on Jun. 30, 2007 at KC, the flood at SYR was on Aug. 13, the former is not only 44 days early, but also extremely big 5000 in Jun. at KC, which was remarkably erred.

The SYR has largest glacier coverage in the UIR so that it contributes the largest rate of the summer meltwater and annual flood. The rate of the annual flood between SYR and KC was 0.41 before 1998, the it was elevated to 0.48 since 1999, which is not resulted from the higher temperature since 1999 because the related mean temperature is the same at 29.2℃before and after 1999. In sum, the flood of UIR is easily erred. The date of annual flood in SYR should be earlier 1 day at least than that in KC because the contributing flood from the KM that flows to KC station needs longer propagating time due to longer distance from the upstream.

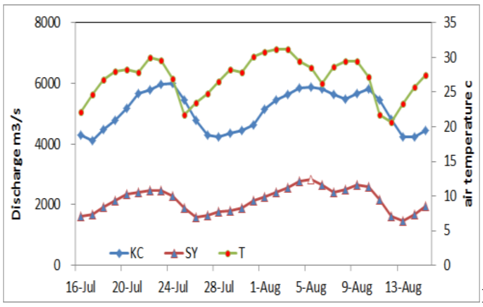

1994

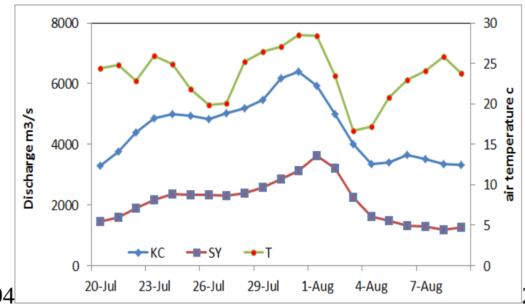

2000

Irrational : the daily graph of annual flood in SYR occurred later than that at KC in 1994 and 2000, KC is at the downstream (T is air temperature at SKD);

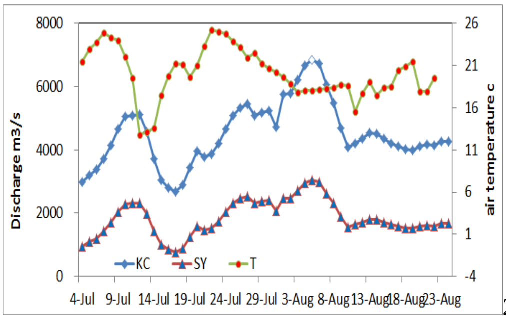

2006

The highest T was much early than the annual floods, and the flood in SYR occurred later than that at KC (△) in 2006.

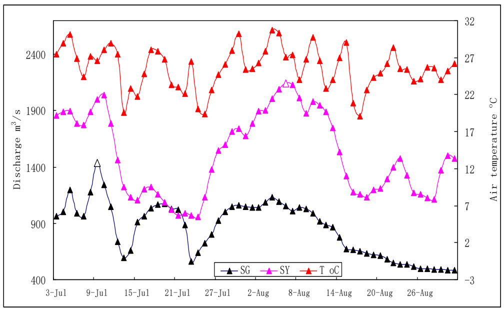

1990

In 1990, the annual flood occurred early on Jul. 9th. (△) in SGR (black line), but on Aug. 6th.(△) in SYR (pink line), another small flood followed in SGR during Jul.14-23, but no in SYR; finally since Aug. 15, discharge gradually declined in SGR, but two small peaks occurred in SYR in 1990.

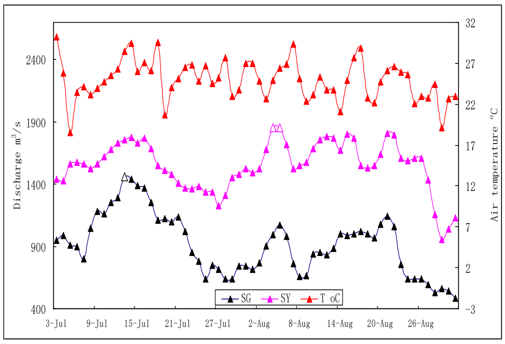

1998

In 1998, the flood occurred early on Jul.13 (△) in SGR (black line), but on Aug. 4 (△) in SYR (pink line).

SI6-Table Monthly data and water budget for KC=SGR+SYR+KM+ungauge

Closure error calculation of monthly flow (in m^3/s) by the equation (1) for the UIR in 1985-1998 (exclude 1993, 1995)

SGR

	Apr	May	Jun	Jul	Aug	Sep
1985	35.2	76.9	201.0	368.5	437.4	230.5
1986	21.6	65.8	182.8	412.5	481.1	234.9
1987	24.5	39.3	112.5	470.0	757.7	433.2
1988	35.4	114.6	317.7	874.0	671.6	406.6
1989	23.4	47.4	185.6	430.4	453.6	263.6
1990	26.5	62.9	524.6	961.5	757.9	327.3
1991	36.8	55.1	375.5	627.1	684.4	434.2
1992	38.6	120.0	312.5	738.7	685.4	298.1
1994	24.6	107.0	485.5	1230.9	1121.9	396.7
1996	42.7	86.9	328.9	585.2	984.9	391.7
1997	27.3	104.1	361.6	1045.7	819.4	308.0
1998	32.0	58.2	282.9	1027.4	824.7	281.1

SYR

	Apr	May	Jun	Jul	Aug	Sep
1985	44.3	73.8	314.6	915.0	1239.6	519.0
1986	41.4	55.6	259.2	1059.0	1106.1	484.8
1987	43.3	58.6	291.7	839.4	1016.1	611.5
1988	56.2	121.6	477.7	1334.0	1352.6	486.2
1989	46.4	81.4	280.8	872.4	773.0	385.8
1990	42.9	206.8	677.0	1474.1	1565.8	700.8
1991	45.7	75.0	619.4	1250.0	1170.9	793.2
1992	38.1	80.4	393.4	962.0	1241.2	613.6
1994	39.2	95.4	482.4	1995.6	1931.3	662.1
1996	53.4	73.5	632.1	1047.2	1403.9	576.6
1997	46.1	84.7	414.9	1579.6	1312.3	622.6
1998	45.8	132.3	440.1	1520.7	1581.0	775.8

KM-stream

	Apr	May	Jun	Jul	Aug	Sep
1985	120.7	244.6	783.7	960.8	929.7	494.0
1986	130.7	327.8	1108.8	1468.3	1170.8	439.1
1987	123.7	239.7	977.3	1318.9	1000.6	506.4
1988	260.0	921.3	1543.6	1847.2	1221.6	518.6
1989	116.8	296.7	1158.3	1801.2	1186.7	484.6
1990	156.7	1297.8	1782.6	1570.6	1196.7	514.0
1991	174.0	383.3	1593.7	1694.2	1062.0	682.8
1992	123.9	397.7	1257.3	1439.2	1086.0	702.2
1993	153.6	599.0	1098.4	1220.2	748.2	475.0
1995	128.2	526.7	1040.8	1124.4	957.2	427.9
1997	144.5	405.4	735.8	1087.4	893.3	644.8

1998	170.7	503.8	1260.8	1842.9	1249.1	690.4

	Apr	May	ungauge Jun	Jul	Aug	Sep
1985	2.4	4.9	15.7	19.2	18.6	9.9
1986	2.6	6.6	22.2	29.4	23.4	8.8
1987	2.5	4.8	19.5	26.4	20.0	10.1
1988	5.2	18.4	30.9	36.9	24.4	10.4
1989	2.3	5.9	23.2	36.0	23.7	9.7
1990	3.1	26.0	35.7	31.4	23.9	10.3
1991	3.5	7.7	31.9	33.9	21.2	13.7
1992	2.5	8.0	25.1	28.8	21.7	14.0
1994	2.6	10.6	22.8	29.8	28.6	12.3
1995	2.6	10.5	20.8	22.5	19.1	8.6
1997	2.9	8.1	14.7	21.7	17.9	12.9
1998	3.4	10.1	25.2	36.9	25.0	13.8

		SG+SY+KM+UNGAUGE				KC						
	Apr	sum	May	sum	Jun	sum	Jul	sum	Aug	sum	Sep	sum
1985	211.9	203.7	553.3	402.6	1604.1	1322.8	2496.1	2273.2	2819.9	2634.6	1338.0	1258.4
1986	235.2	197.6	518.8	459.1	1562.5	1584.1	3075.1	2983.8	2745.4	2793.2	1282.8	1171.9
1987	218.2	195.2	362.6	344.7	1509.1	1410.9	2764.8	2667.9	2832.8	2804.4	1680.9	1566.3
1988	481.4	359.4	1347.6	1185.2	2514.5	2385.3	4215.4	4110.6	3529.7	3282.4	1733.3	1427.0
1989	221.1	190.1	603.8	434.4	2213.8	1659.4	3446.0	3158.0	2879.5	2448.9	1281.8	1148.5
1990	225.9	230.9	1859.2	1606.4	3255.3	3037.7	4101.9	4053.3	3921.7	3556.3	2048.9	1557.5
1991	254.9	261.7	540.3	524.9	2860.1	2636.4	3781.0	3622.1	3207.0	2949.3	2060.9	1930.7
1992	249.8	204.2	674.9	610.0	2186.2	2000.9	3385.0	3183.1	3255.9	3045.1	1706.5	1635.0
1994	208.8	197.7	954.2	745.9	2601.2	2140.2	5129.1	4762.0	4848.8	4525.3	1949.8	1692.3
1996	339.3	307.2	753.5	540.7	3183.7	2502.9	3791.1	3114.0	4159.6	3733.8	1700.1	1579.5
1997	234.9	222.2	652.0	606.4	1764.2	1534.4	3894.6	3745.4	3254.8	3051.8	1573.9	1594.7
1998	247.8	253.7	867.7	709.4	2090.2	2021.6	4547.2	4446.3	3813.0	3692.2	2006.2	1747.4

SI6-Table 3Estimated monthly bias during Apr. to Sep.

	KC/Sum ratio Apr	KC/Sum ratio May	KC/Sum ratio Jun	KC/Sum ratio Jul	KC/Sum ratio Aug	KC/Sum ratio Sep	Mean/May-Sep
1985	1.047	1.383	1.220	1.103	1.074	1.068	
1986	1.198	1.138	0.993	1.036	0.987	1.099	
1987	1.125	1.059	1.077	1.041	1.014	1.077	
1988	1.349	1.146	1.061	1.030	1.079	1.219	
1989	1.171	**1.399**	1.343	1.097	1.182	1.121	1.229
1990	0.985	1.167	1.078	1.016	1.106	1.320	
1991	0.981	1.037	1.091	1.049	1.091	1.071	
1992	1.231	1.114	1.099	1.068	1.073	1.048	
1994	1.063	1.288	1.222	1.080	1.075	1.156	
1996	1.112	**1.403**	1.280	1.223	1.118	1.080	1.221
1997	1.064	1.082	1.155	1.043	1.070	0.991	
1998	0.984	1.232	1.040	1.027	1.036	1.148	
mean (%)	1.109	1.204	1.138	1.068	1.075	1.117	1.120

The data marked in red mean the maximum extreme biases.

SI7-Fig. information on hydrometric sections and the cable-sinker.
Kachura, Shyok and Shigar River at its Discharge Point

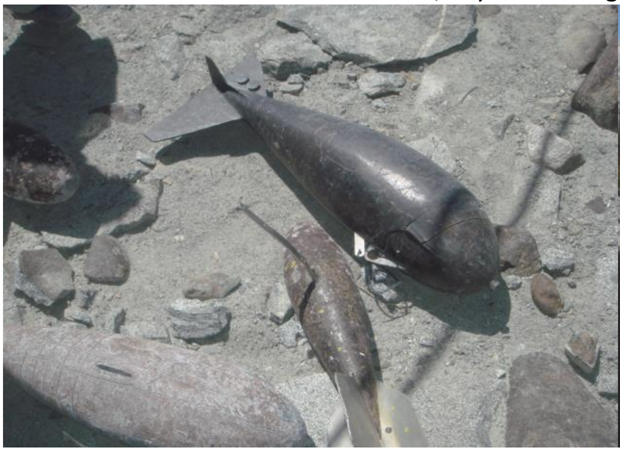

Made of Pb-fish meter at KC (by Liu)

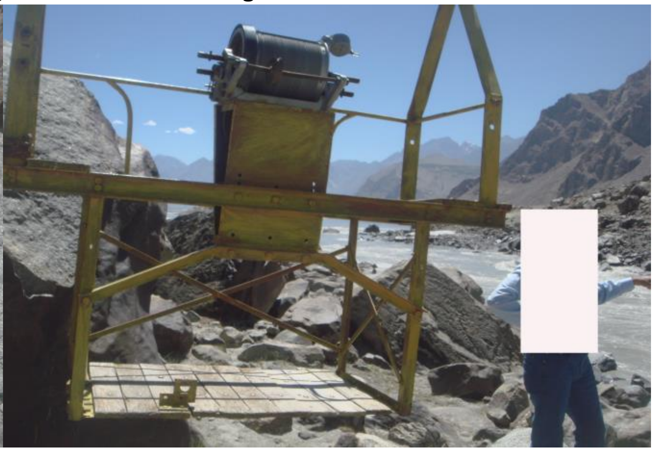

Cable meter at KC (by Liu)

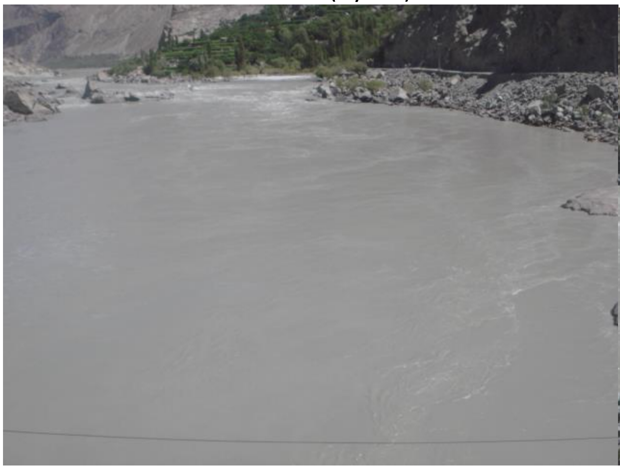

Hydrometric section at SYR (by Liu)

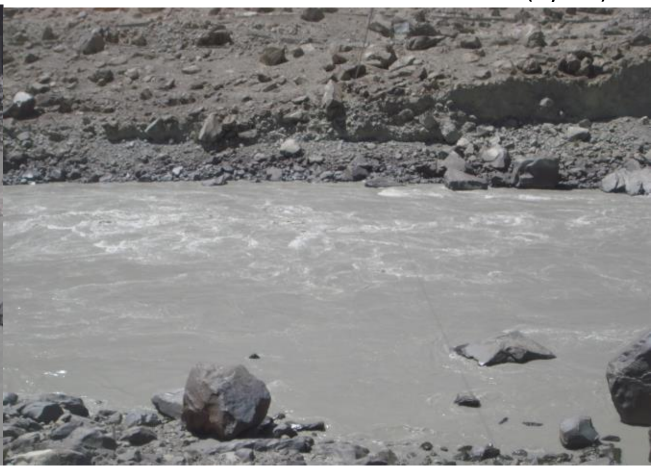

Hydrometric section at KC (by Liu)

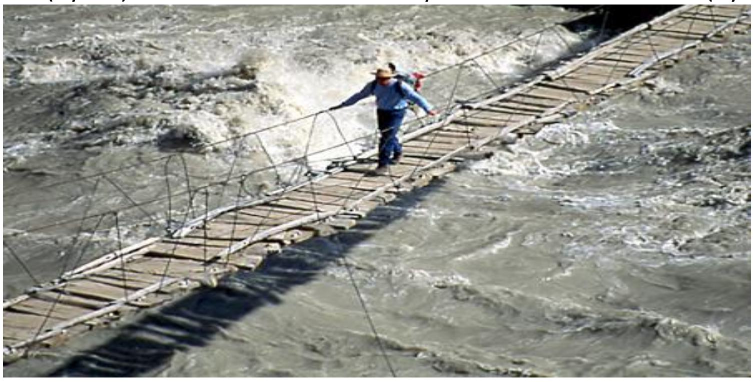

Unsteady flow at SYR bridge (by Liu)

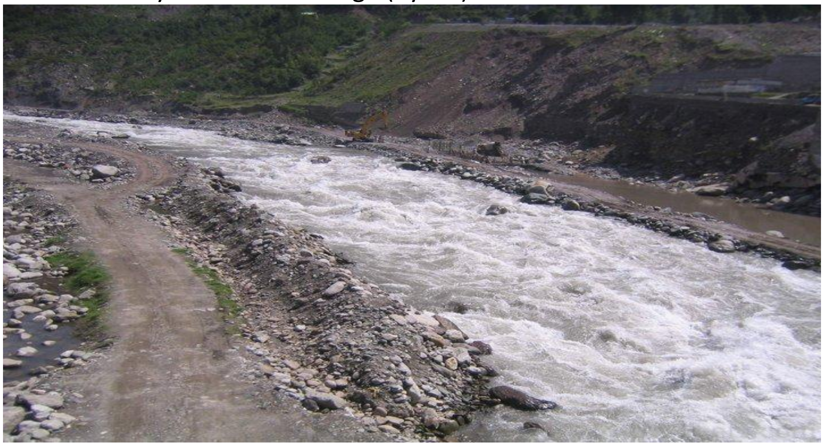

Unsteady river flow at KM-stream section (by Liu)

SI9 References

SI8- references

(1) Li J., et al., Investigation and study of the Batura Glacier in the Hunza valley, Pakistan, 110-126, Sci. Press., Beijing (1980)

(2) Qamar, ul. Zaman., J., Liu. Mass balance of Siachen Glacier, Nubra valley, Karakoram Himalaya: facts or flaws? J. Glacio.,doi: 10. 3189/ 2015JoG15J120 (2015).

(3) Wang, D., J.S. Liu, LJ., Hu, M. Zhang. Monitoring and analysis to glacier lake outburst flood and dammed lake from the upper Yarkant River, the China-Karakoram, J. Glacio. & Geocryo., 31(5),808-814 (2009).

(4) Yogendra Prasad, Dumkhar HE Project 3x15 MW, Preliminary Feasibility Report, NHPC, India (2004).

(5) Danial, Hashmi, Flow routing model for the Upper Indus River (Pakistan), University of British Columbia, Canada (1994).

(6) Minora, U, D. Bocchiola, C. D'Agata, et al. 2001–2010 glacier changes in the Central Karakoram National Park: a contribution to evaluate the magnitude and rate of the "Karakoram anomaly", The Cryosphere Discuss., 7, 2891–2941(2013).

(7) Dymond, J.R. & R., Christan. Accuracy of discharge determined from a rating curve, Hydrological Sciences-Journal, 4,12-19 (1982).

(8) Bocchiola, D. & G. Diolaiuti. Recent (1980–2009) evidence of climate change in the upper Karakoram, Pakistan. Theor Appl Climatol., 113: 611–641 (2013).

(9) Asif Khan, et al. How large is the Upper Indus Basin? The pitfalls of auto-delineation using DEMs. J. Hydro., http://dx.doi.org/ 10.1016/j. jhydrol.2013.11.028 (2013).

(10) Laurence, C., et al., Estimation of river discharge, propagation speed, and hydraulic geometry from space: Lena River, Siberia. Water resources research, 44,W03427,doi:0.1029/2007WR006133 (2008).

(11) Montanari, A, & G Grossi. Estimating the uncertainty of hydrological forecasts: A statistical approach, Water Resources Research, 1-9 (2008)

(12) Dottori F, et al., A dynamic rating curve approach to indirect discharge Measurement. Hydrol. Earth Syst. Sci., 13, 847–863 (2009).

(13) Yu, B. A systematic over-estimation of flows. J. Hydrol., 233:258-262 (2000).

(14) Jose-Luis Guerrero, et al. Temporal variability in stage–discharge relationships. J. Hydrol., 446–447, 90–102 (2012).

(15) Thayyen, RJ., & J. T. Gergan. Role of glaciers in watershed hydrology: a preliminary study of a Himalayan catchment, The Cryosphere, 4, 115–128 (2010).

(16) Karki M.B., A.B. Shrestha, M. Winiger. Enhancing Knowledge Management and Adaptation Capacity for Integrated Management of Water Resources in the Indus River Basin, Mountain Res. and Deve., DOI: 10.1659/MRD-JOURNAL–D-11-00017.1 (2011).

(17) Agarwal, V., T. Bolch, T.H. Syed, et al. Nagaich. Area and mass changes of Siachen Glacier (East Karakoram). J. Glacio., doi: 10.1017/jog.2016.127 (2016).
